# 2-Oxoglutarate regulates binding of hydroxylated hypoxia-inducible factor to prolyl hydroxylase domain 2†
†Electronic supplementary information (ESI) available: Details of experimental procedures and additional experiments. See DOI: 10.1039/c8cc00387d


**DOI:** 10.1039/c8cc00387d

**Published:** 2018-03-09

**Authors:** Martine I. Abboud, Tom E. McAllister, Ivanhoe K. H. Leung, Rasheduzzaman Chowdhury, Christian Jorgensen, Carmen Domene, Jasmin Mecinović, Kerstin Lippl, Rebecca L. Hancock, Richard J. Hopkinson, Akane Kawamura, Timothy D. W. Claridge, Christopher J. Schofield

**Affiliations:** a Chemistry Research Laboratory , University of Oxford , 12 Mansfield Road , Oxford , OX1 3TA , UK . Email: tim.claridge@chem.ox.ac.uk ; Email: christopher.schofield@chem.ox.ac.uk; b School of Chemical Sciences , The University of Auckland , New Zealand; c Department of Chemistry , Britannia House , Kings College London , UK; d Department of Chemistry , University of Bath , Claverton Down , Bath , BA2 7AY , UK; e Institute for Molecules and Materials , Radboud University , Heyendaalseweg 135 , 6525 AJ Nijmegen , The Netherlands; f Leicester Institute of Structural and Chemical Biology and Department of Chemistry , University of Leicester , Leicester , LE1 7RH , UK

## Abstract

Prolyl hydroxylation of hypoxia inducible factor (HIF)-α, as catalysed by the Fe(ii)/2-oxoglutarate (2OG)-dependent prolyl hydroxylase domain (PHD) enzymes, has a hypoxia sensing role in animals. We report that binding of prolyl-hydroxylated HIF-α to PHD2 is ∼50 fold hindered by prior 2OG binding; thus, when 2OG is limiting, HIF-α degradation might be inhibited by PHD binding.

## Abstract 2

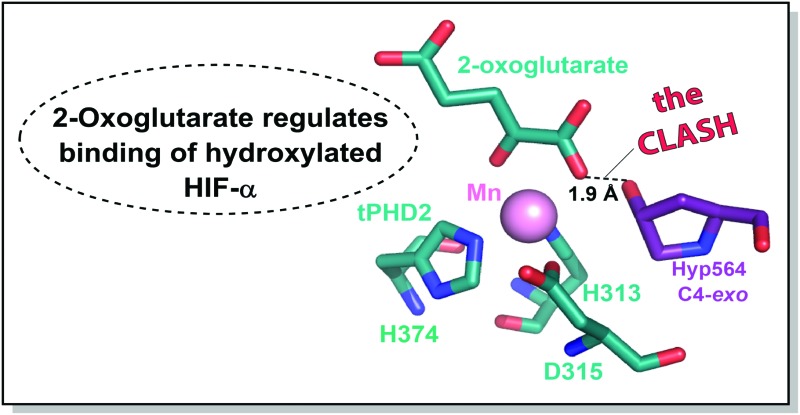
The binding of prolyl-hydroxylated HIF-α to PHD2 is hindered by prior 2OG binding; likely, leading to the inhibition of HIF-α degradation under limiting 2OG conditions.

## 

The chronic response to limiting O_2_ in animals involves upregulation of multiple genes as enabled by the α,β-heterodimeric hypoxia inducible factors (HIFs). Under normoxia, HIF-α subunits are efficiently degraded by proteasomes; in hypoxia, HIF-α subunits accumulate, so enabling the α,β-HIF complex to promote expression of HIF target genes, including genes for biomedicinally important proteins, such as the vascular endothelial growth factor and erythropoietin.^1^ There is thus a considerable interest in the therapeutic manipulation of the HIF system (Fig. S1, ESI†).^2^,^3^ *trans*-4-Prolyl hydroxylation of HIF-α substantially (∼10^3^ fold) increases the strength of its binding to the von Hippel–Lindau protein (pVHL), the targeting component of a ubiquitin E3 ligase.^4^–^7^ HIF-α prolyl hydroxylation occurs at P402 and P564 (HIF-1α) in the N- and C-terminal oxygen dependent degradation domains (NODD/CODD, respectively), and is catalysed by human Fe(ii)- and 2-oxoglutarate (2OG)-dependent oxygenases (PHD1-3),^8^,^9^ the most important of which is likely PHD2 (Fig. S2, ESI†).^10^ Evidence from studies with proteins, cells, and animals supports the proposal that PHD activity is limited by O_2_ availability. PHD catalysis involves binding of 2OG, HIF-α, then O_2_, to the active site, with CO_2_ and succinate being produced as coproducts (Fig. S3, ESI†).^11^–^13^ Evidence has been presented that the PHDs act as hypoxia sensors, including by the manifestation of an unusually slow reaction with O_2_.^11^,^12^ PHD catalysis has the potential to be regulated by Fe(ii) and 2OG availability, as well as by succinate and other TCA cycle intermediates.^14^,^15^ C4 *trans* prolyl hydroxylation increases the fraction of the C4-*exo* prolyl conformation, as observed when HIF-α is complexed with pVHL,^4^–^7^,^16^ likely in part due to the ‘*gauche*’ stereoelectronic effect.^17^ The interactions of HIF-α with pVHL and the PHDs are of interest from a chemical perspective because of the profound effect of hydroxylation on biological function. Here, we report evidence that the presence of the 2OG cosubstrate at the PHD2 active site can regulate binding of unhydroxylated *versus* prolyl hydroxylated HIF-α (Fig. 1).

**Fig. 1:**
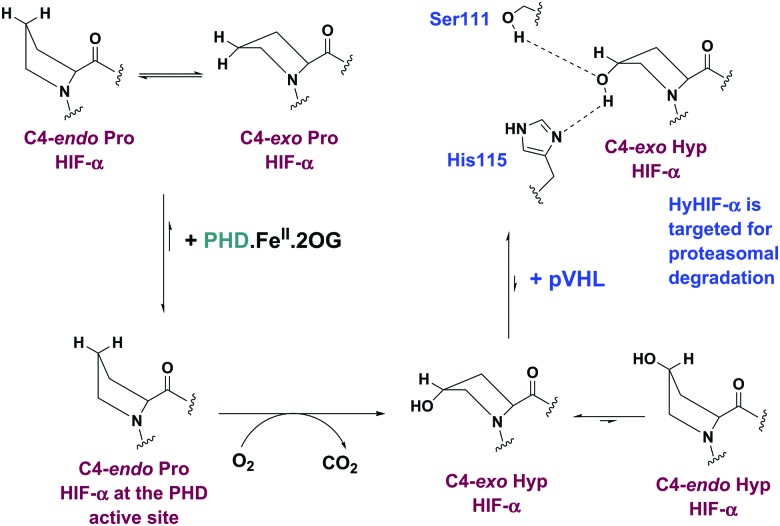
Outline role of PHD-catalysed HIF-α hydroxylation.

We initially investigated the binding of 19-mer HIF-1α CODD and hydroxylated CODD (hyCODD) peptides to recombinantly expressed PHD2_181–426_ (tPHD2) using non-denaturing mass spectrometry (ESI-MS).^18^,^19^ The results indicated that CODD binds strongly not only to tPHD2·Fe, but also to *apo*-tPHD2 (Fig. 2). Addition of 2OG to a 1 : 1 mixture of *apo*-tPHD2·CODD did not lead to observation of an *apo*-tPHD2·2OG complex, consistent with 2OG binding to the Fe(ii) in catalysis.^20^ Analysis of an equimolar mixture of *apo*-tPHD2, Fe(ii), 2OG, and CODD manifested masses corresponding to complexes of tPHD2·Fe·CODD-OH and tPHD2·Fe·CODD-OH·succinate, but not any observable quaternary complex with 2OG (Fig. 2). These results indicate that CODD and hyCODD bind to *apo*-tPHD2/tPHD2·Fe (Fig. 2).

**Fig. 2:**
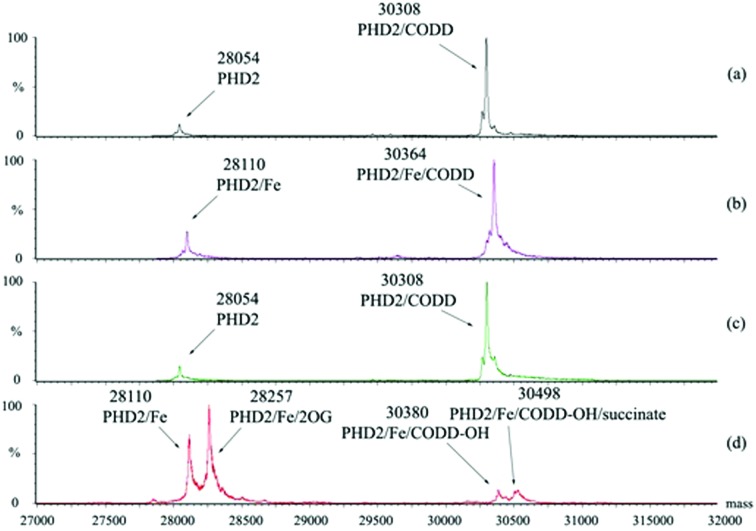
Deconvoluted non-denaturing ESI-MS spectra showing CODD substrate, Fe(ii) cofactor, and 2OG cosubstrate binding to tPHD2. (a) Black trace: 1 : 1, tPHD2 : CODD; (b) purple trace: 1 : 1 : 1, tPHD2 : Fe : CODD; (c) green trace: 1 : 1 : 1, tPHD2 : 2OG : CODD; (d) red trace: 1 : 1 : 1 : 1, tPHD2 : Fe : 2OG : CODD. For assay conditions, see Materials and methods.

Having shown that the interaction between tPHD2 and CODD is preserved in the gas phase, we then carried out solution studies. NMR studies^21^,^22^ using ^15^N-labelled PHD2_181–402_ showed that CODD and hyCODD bind with approximately equal affinity to *apo*-PHD2_181–402_ and PHD2_181–402_·Zn (*i.e.* both saturated *apo*-PHD2_181–402_ and PHD2_181–402_·Zn at 2.7-fold excess) (Fig. 3A and B). The spectrum of the PHD2_181–402_·Zn·2OG·CODD complex was observed as reported.^22^ By contrast, hyCODD only showed weak binding to PHD2_181–402_·Zn·2OG (Fig. 3C). The ^1^H–^15^N HSQC spectra of *apo*-PHD2_181–402_·hyCODD and *apo*-PHD2_181–402_·2OG·hyCODD are similar in the presence of excess hyCODD (Fig. 3D). The overall results imply hyCODD binding is blocked by the presence of 2OG; as anticipated, binding of CODD to PHD2·Zn is not hindered by 2OG.^22^,^23^

**Fig. 3:**
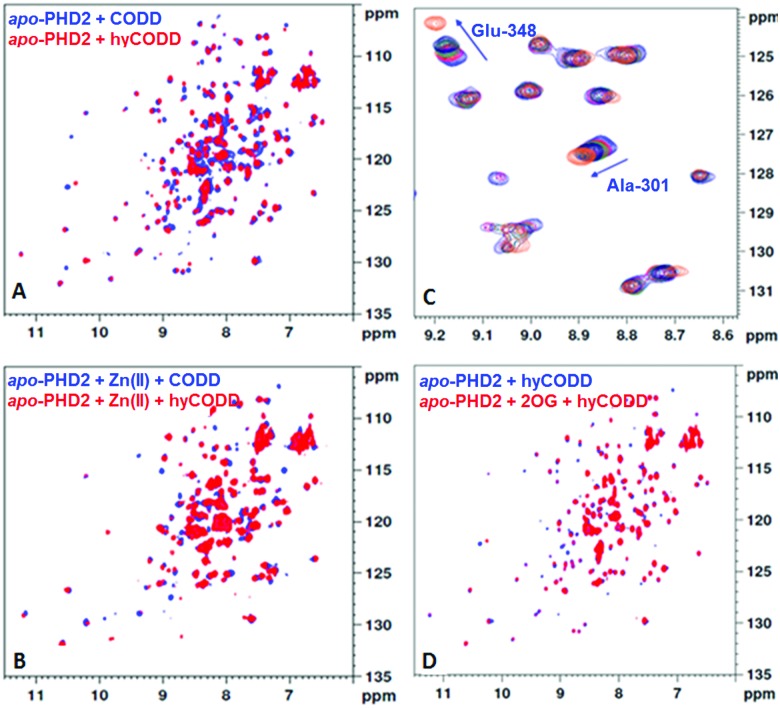
^1^H–^15^N HSQC binding studies reveals 2OG hinders binding of hyCODD, but not CODD, to PHD2·Zn(ii). (A) Overlays of ^1^H–^15^N HSQC spectra for *apo*-^15^N-PHD2_181–402_·CODD (blue) and *apo*-^15^N-PHD2_181–402_·hyCODD (red); mixture: 150 μM *apo*-^15^N-PHD2_181–402_, 700 μM CODD or 550 μM hyCODD. (B) Overlays of ^1^H–^15^N HSQC spectra for ^15^N-PHD2_181–402_·Zn·CODD (blue) and ^15^N-PHD2_181–402_·Zn·hyCODD (red); mixture: 150 μM *apo*-^15^N-PHD2_181–402_, 300 μM Zn(ii), 400 μM CODD/hyCODD. (C) Overlays of a region of the ^1^H–^15^N HSQC spectra for ^15^N-PHD2_181–402_·Zn·2OG with 0 μM hyCODD (blue), 75 μM hyCODD (red), 112.5 μM hyCODD (green), 150 μM hyCODD (purple) and 300 μM hyCODD (orange). Mixture: 50 μM *apo*-^15^N-PHD2_181–402_, 100 μM Zn(ii), 50 μM 2OG. (D) Overlays of the ^1^H–^15^N HSQC spectra for a mixture of 150 μM *apo*-^15^N-PHD2_181–402_ and 400 μM hyCODD (blue) and a mixture of 50 μM *apo*-^15^N-PHD2_181–402_, 300 μM 2OG, 400 μM hyCODD (red). Buffering was with 50 mM Tris-D_11_, pH 6.6, in 95% H_2_O, 5% D_2_O.

To investigate the relative affinities of hyCODD and CODD to tPHD2, a fluorescence polarisation assay with N-terminal fluorescein-tagged CODD (CODD*) was developed (Fig. S4, ESI†). Consistent with the NMR and MS data, CODD and hyCODD bind to *apo*- and metallated-tPHD2 equally strongly (within a 2-fold difference). With tPHD2·Zn·2OG, hyCODD was observed to bind ∼50-fold less strongly than CODD (Fig. S5, ESI†). Isothermal calorimetry results showed that both CODD and hyCODD bind with similar affinity to *apo*-tPHD2 (9.4 ± 4.5 and 4.6 ± 1.4 μM, respectively); by contrast, CODD, but not hyCODD, was observed to bind to tPHD2·Zn·2OG (*K*_D_ = 1.8 ± 0.4 μM) (Fig. S6, ESI†). Addition of hyCODD displaced 2OG from tPHD2·Zn·2OG as observed by NMR, implying hyCODD and 2OG compete for binding to the metal complexed tPHD2 (Fig. S7, ESI†). CODD does not displace 2OG, consistent with the FP observations (Fig. S5, ESI†) and the binding requirements of the catalytic cycle (Fig S2, ESI†).

Mutation of genes encoding for TCA cycle enzymes are observed in tumours with consequent effects on metabolite levels, resulting in proposed inhibition of human 2OG oxygenases, including the PHDs, so promoting tumorigenesis.^14^,^15^ The effects of TCA cycle intermediates (Fig. S8, ESI†) on CODD/hyCODD binding to tPHD2·Zn were investigated by NMR; of the tested compounds, only fumarate and succinate were observed to bind to tPHD2·Zn, consistent with previous reports.^15^ By contrast with 2OG, the binding of fumarate or succinate was only weakly disrupted by hyCODD (Fig. S9, ESI†), implying the ‘additional’ carbonyl of 2OG has a role in hindering hyCODD, but not CODD, binding to the tPHD2·metal complex. The observation of simultaneous binding of succinate and hyCODD to tPHD2 is consistent with the MS results (Fig. 2d).

Analysis of crystallographic data of tPHD2·HIF-α complexes indicates the metal chelated 2OG C1 carboxylate may hinder hyCODD, but not CODD, binding (Fig. 4). By contrast, modelling of succinate binding (using tPHD2 structures and those of succinate complexed with other 2OG oxygenases)^15^,^22^ indicates that it will not manifest a steric clash with hyCODD (Fig. S10, ESI†). Crystallographic analysis implies that in the tPHD2·Mn·2OG·ODD complexes, the substrate prolyl ring adopts the C4-*endo* conformation which changes to the C4-*exo* on hydroxylation.^4^,^5^,^17^ This proposal is supported by the reported observation that *cis*, but not *trans*, 4-fluoro prolyl CODD is a tPHD2 substrate^17^ and by the ^19^F NMR studies which show a lack of efficient binding of *trans*-4-fluoro prolyl CODD to tPHD2·Fe·2OG (Fig. S11, ESI†). Modelling studies imply the C4-*exo*, rather than the C4-*endo*, conformation of *trans*-4-prolyl hydroxylated CODD will lead to a clash between the hyCODD alcohol and the 2OG C1 carboxylate (Fig. S12–S18, ESI†).

**Fig. 4:**
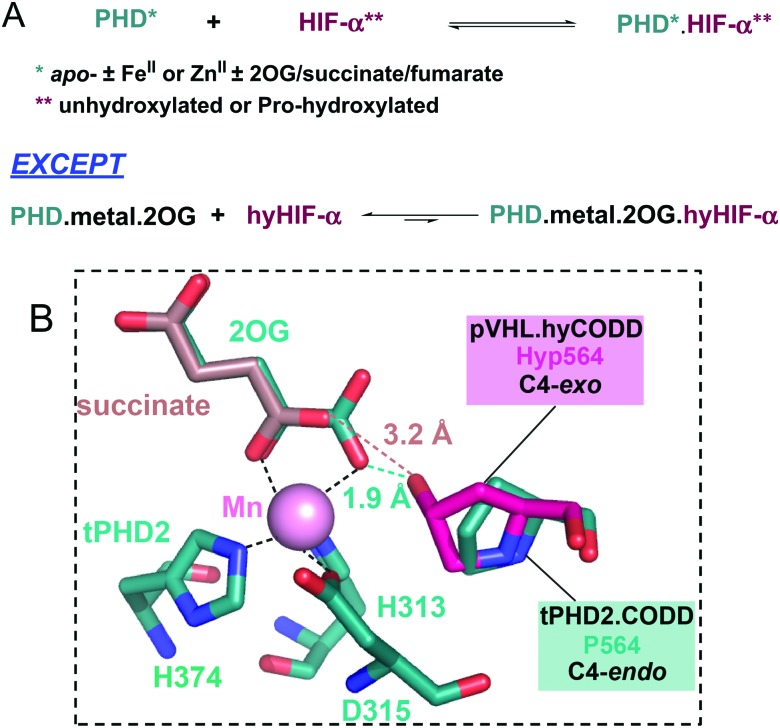
Summary of PHD-HIF/hyHIF-α binding results, highlighting the proposed role of 2OG in blocking hyHIF-α binding. (A) HIF and hydroxylated HIF (hyHIF) bind equally strongly to *apo*-tPHD2 and metallated tPHD2; 2OG differentiates between the binding of HIF and hyHIF. (B) View from a structure of tPHD2·Mn·2OG·CODD complex (PDB ID: ; 5L9B).^22^ The view was overlaid with the hyCODD conformation (fuchsia) as bound to pVHL (PDB ID: ; 1LQB)^5^ showing the potential steric clash between the 2OG C1 carboxylate and the C4-e*xo* hydroxyl group in hyCODD (1.9 Å). No steric clash is predicted between the carboxylate of succinate and the C4-*exo* hydroxyl group in hyCODD (3.2 Å). The succinate (light brown) binding mode was based on 2OG binding as in PDB ID: ; 5L9B.^22^

Whether hyCODD competes with 2OG and/or NODD was investigated using 1D CLIP HSQC (with selective ^13^C-inversion) NMR using ^13^C-2OG and ^13^C-NODD.^24^,^25^ The addition of unlabelled CODD to tPHD2·Zn·2OG·NODD leads to NODD, but not 2OG, displacement, suggesting that the affinity of CODD to tPHD2 is higher than NODD, consistent with previous work.^12^,^22^ Addition of hyCODD manifests displacement of 2OG and NODD (Fig. S19, ESI†), implying competition with both. Inhibition of the tPHD2-catalysed 2OG turnover to succinate was observed by ^1^H NMR in the presence of hyCODD with CODD as a substrate and, to a greater extent, with NODD as a substrate (Fig. S20, ESI†).

The results reveal 2OG binding hinders the binding of hyCODD, but not CODD/NODD, to the tPHD2·metal·2OG complex. Thus, competition with 2OG can promote release of prolyl hydroxylated HIF-1α from tPHD2 (and by implication other PHD/HIF-α isoform combinations), so enabling HIF-α degradation.^4^,^5^ Once the *trans*-4-hydroxyproline is formed, the ‘*gauche*’ stereoelectronic effect biases the conformation towards the C4-*exo* form, as observed in the hyCODD·pVHL complex.^4^,^5^,^17^ Formation of the C4-*exo* conformation at the tPHD active site may thus promote a clash between the hyCODD alcohol and the 2OG C1 carboxylate, which is involved in Fe(ii) chelation (Fig. S10, ESI†).

Hydroxylated prolyl HIF (hyHIF)-α is upregulated in many tumour cells.^26^ Under cellular circumstances when there is accumulation of hyHIF-α (*e.g.* due to mutations to the gene encoding for pVHL as occurs in the VHL disease),^27^,^28^ or if iron (*e.g.* in anaemia) or 2OG availability is limiting (*e.g.* potentially due to mutations in the genes encoding for isocitrate dehydrogenase),^29^,^30^ cellular formation of the PHD·Fe·hyHIF-α or PHD·hyHIF-α complexes may become substantial, with consequent potential limitation of the HIF-mediated hypoxic response. The results thus suggest a negative feedback mechanism for PHD activity, which is linked to TCA cycle metabolism.

We thank the Biochemical Society (Krebs Memorial Award), the Wellcome Trust (106244/Z/14/Z), Cancer Research UK (C8717/A18245), the British Heart Foundation Centre of Research Excellence, Oxford (RE/13/130181), the John Fell Fund (143/075), a Junior Research Fellowship from Kellogg College, Oxford, and the Biotechnology and Biological Sciences Research Council for funding our work. We thank EPSRC and PRACE for providing computational resources through access to Archer and other European HPC facilities.

## Conflicts of interest

The authors declare no conflicts of interest.

## Supplementary Material

Supplementary information
