# Supplementary material for: 2-Oxoglutarate regulates binding of hydroxylated hypoxia-inducible factor to prolyl hydroxylase domain 2
Source: Chem Commun (Camb). 2018 Mar 9;54(25):3130–3. doi: 10.1039/c8cc00387d (PMC5885369; doi:10.1039/c8cc00387d)
Supplement: Supplementary file 1 [file CC-054-C8CC00387D-s001.pdf]

Electronic Supplementary Material (ESI) for ChemComm.
This journal is © The Royal Society of Chemistry 2018

2-Oxoglutarate Regulates Binding of Hydroxylated Hypoxia-Inducible Factor to Prolyl Hydroxylase Domain 2

Martine I. Abboud,[a] Tom E. McAllister,[a] Ivanhoe K.H. Leung,[a,b] Rasheduzzaman Chowdhury,[a] Christian Jorgensen,[c] Carmen Domene,[a,d] Jasmin Mecinović,[a,e] Kerstin Lippl,[a] Rebecca L. Hancock,[a] Richard J. Hopkinson,[a,f] Akane Kawamura,[a] Timothy D.W. Claridge,[a]† and Christopher J. Schofield[a]†

[a.] *Chemistry Research Laboratory, University of Oxford, 12 Mansfield Road, Oxford, OX1 3TA, U.K.*
[b.] *Department of Chemistry, Britannia House, Kings College London, U.K.*
[c.] *School of Chemical Sciences, University of Auckland, New Zealand.*
[d.] *Department of Chemistry, University of Bath, Claverton Down, Bath, BA2 7AY, U.K.*
[e.] *Institute for Molecules and Materials, Radboud University, Heyendaalseweg 135, 6525 AJ Nijmegen, The Netherlands.*
[f.] *Department of Chemistry, University of Leicester, Leicester, LE1 7RH, U.K.*
† Address correspondence to tim.claridge@chem.ox.ac.uk and christopher.schofield@chem.ox.ac.uk

Contents

Materials and Methods *5*

Reagents 5

Peptides 5

Protein Production 5

Non-Denaturing MS Experiments 6

Fluorescence Polarisation Assay 6

Isothermal Titration Calorimetry Experiments 7

NMR Experiments 8

CPMG-edited ^{1}H NMR Experiments 8

2OG Displacement Monitoring by CPMG-edited ^{1}H NMR 8

Binding of TCA Cycle Intermediates to tPHD2 by CPMG-edited ^{1}H NMR 8

TCA Cycle Intermediates Displacement Monitoring by CPMG-edited ^{1}H NMR 9

^{1}H-^{15}N HSQC Experiments 9

^{13}C-2OG and ^{13}C-NODD/CODD Displacement Experiments 9

2OG Turnover Monitoring by ^{1}H NMR 9

^{19}F NMR Monitoring of Labelled Peptides 10

Docking 10

Computational Studies 11

MD Simulation Studies 11

QM/MM Studies 12

Supplementary Figures *14*

Fig. S1. Outline of the machinery involved in the cellular response to hypoxia in animals. 14

Fig. S2. Outline mechanism for PHD2 catalysed HIF-α hydroxylation. 15

Fig. S3. View from a crystal structure of tPHD2 in complex with metal, 2-oxoglutarate (2OG), and CODD (PDB ID: 5L9B)...........16

Fig. S4. Fluorescence polarisation studies of the binding of CODD* to tPHD2...........17

Fig. S5. Fluorescence polarisation studies on the binding of CODD and hyCODD to tPHD2...........18

Fig. S6. Isothermal titration calorimetry (ITC) studies on the binding of CODD and hyCODD to (A) *apo*-tPHD2 and (B) tPHD2.Zn.2OG...........19

Fig. S7. Investigations of the binding mode of hyCODD to tPHD2.Zn using CPMG-edited ^{1}H NMR 2OG displacement analyses...........20

Fig. S8. Structures of the TCA cycle intermediates tested for binding to tPHD2.Zn...........20

Fig. S9. Investigations of the binding of TCA cycle intermediates to tPHD2.Zn in the presence of Zn(II) and hyCODD by CPMG-edited ^{1}H NMR analyses...........21

Fig. S10. Views derived from tPHD2 crystal structures evaluating potential clashes between metal complexed 2OG or succinate carboxylate and the prolyl alcohol of hyCODD...........22

Fig. S11. ^{19}F NMR monitoring of fluoride release by tPHD2 using ^{19}F-labelled *cis* or *trans* P564 CODD as a substrate...........23

Fig. S12. The observed tPHD2 backbone RMSD (Å) in MD simulations of tPHD2 complexes..........24

Fig. S13. The observed active site RMSD (Å) in MD simulations of tPHD2 complexes..........24

Fig. S14. The observed D315-H564 distance ($O_{\delta1}$-Hε) (Å) in MD simulations of tPHD2 complexes...........25

Fig. S15. The observed backbone RMSD (Å) using MD simulations of tPHD2.Fe.2OG complexed with (a) CODD *cis*-Hyp564 or (b) CODD *trans*-Hyp564...........25

Fig. S16. The observed tPHD2 backbone RMSD (Å) using simulations of tPHD2.Fe complexed with (a) CODD *cis*-Hyp564 or (b) CODD *trans*-Hyp564...........26

Fig. S17. Views of the tPHD2 active site as observed in snapshots at the RMSD plateau from the classical MD production runs 26

Fig. S18. Views of the tPHD2 active site as observed in QM/MM minimised snapshots. 27

Fig. S19. 1D CLIP-HSQC (with selective ^{13}C-inversion) analyses of the binding of hyCODD to the tPHD2.Zn.2OG.NODD complex. 28

Fig. S20. Monitoring of tPHD2 catalysed 2OG to succinate turnover in the presence of added hyCODD as observed by ^{1}H NMR using either CODD or NODD as a substrate. 29

***Supplementary Tables*...30**

Table S1. Summary of system setup and calculations. 30

Table S2. QM/MM energy minimisation results. 30

***References* 31**

Materials and Methods

Reagents

2-Oxoglutarate disodium salts (2OG), L-sodium ascorbate (ascorbate), $FeSO_4 \cdot 7H_2O$, and zinc sulfate were from Sigma Aldrich.

Peptides

HIF-1α CODD (DLDLEMLAPYIPMDDDFQL-NH_2), hydroxylated CODD (hyCODD) (DLDLEMLAHypYIPMDDDFQL-NH_2), ^{19}F-labelled *cis* or *trans* CODD (DLDLEMLAP*YIPMDDDFQL-NH_2), and NODD (EELAQLAPTPGDAIISLDF-NH_2) peptides were from Severn Biotech. All peptides were prepared as *C*-terminal amides. Synthetic hydroxylated CODD is the same as the enzymatically produced hydroxylated CODD.[1, 2] The fluorescent tracer used (CODD*) was an *N*-terminally fluorescein isothiocyanate (FITC)-labelled HIF-1α CODD peptide, sequence: FITC-DLDLEMLAPYIPMDDDFQL-NH_2; synthesised by GL Biochem (Shanghai) Ltd., China.

Protein Production

The catalytic domain of human $PHD2_{181\text{-}426}$ (tPHD2) was produced and purified as reported.[1] tPHD2 was used for all experiments, unless otherwise stated. For ^{1}H-^{15}N HSQC experiments, a *C*- and *N*-terminal truncated construct of the catalytic domain of PHD2 ($PHD2_{181\text{-}402}$) was used in order to minimise spectral overlap and to reduce transverse relaxation losses to a minimum. [^{15}N]-enriched $PHD2_{181\text{-}402}$ was produced in glucose-enriched minimal medium (M9 salts using 10 g/L of glucose). Protein production was induced by addition of 200 μM isopropyl β-D-1-thiogalactopyranoside (IPTG) when the cell density reached an optical density (OD_{600}) of 0.6–0.8 and left for overnight incubation at 28 °C. Protein purification was as reported.[3, 4] *Apo*-PHD2 was prepared by incubating tPHD2 or $PHD2_{181\text{-}402}$ (2-3 mg/mL) with 15,000-equivalents of EDTA at 4 ºC overnight. The treated

proteins were further purified using a Superdex S200 column (300 mL). The purity of the resulting fractions was ascertained to be > 90 % by SDS-PAGE. Removal of the metal was confirmed by non-denaturing mass spectrometry and activity analyses.

Non-Denaturing MS Experiments

For non-denaturing MS measurements, spectra were obtained using a Waters Q-TOF mass spectrometer (Q-TOF micro, Micromass) with a chip voltage of 1.70 kV ± 0.2 kV and a delivery pressure of 0.25 psi (1 psi = 6.89 kPa) using a NanoMate HD Robot chip-based nanoelectrospray device (Advion Biosciences). Calibration and sample acquisitions were performed in the positive ion mode in the range of 500–5000 *m/z*. Typically, protein samples were sprayed at a cone voltage of 80 V with acquisition/scan times of 10 s/1 s. The pressure at the interface between the atmospheric source and the high vacuum region was 6.60 mbar. Data were processed using MASSLYNX 4.0 (Waters).[5, 6] tPHD2 solutions were desalted using a BioSpin 6 column (Bio-Rad, Hemel Hempstead, UK) into 15 mM ammonium acetate buffer (pH 7.5). Assay mixtures contained 15 µM tPHD2, 15 µM $FeSO_4 \cdot 7H_2O$, 15 µM 2OG, 15 µM CODD buffered with 15 mM ammonium acetate, pH 7.5.

Fluorescence Polarisation Assay

Fluorescence polarisation[7] assays used a PHERAstar FS reader (excitation 485 nm, emission 520 nm). Experiments with *apo*-tPHD2 were conducted in 50 mM Tris-HCl pH 7.5 with 3 µM *apo*-tPHD2 and 5 % (*v*/*v*) DMSO. For experiments with Zn, 1.5 µM *apo*-tPHD2 was used with the addition of $ZnSO_4.7H_2O$ at a final concentration of 50 µM. For experiments with Zn and 2OG, 0.5 µM *apo*-tPHD2 was used with the addition of Zn(SO4)2.7H2O at final concentration of 50 µM and 2OG at a final concentration of 100 µM. tPHD2 was titrated into fixed concentrations of the fluorescent tracer and the changes in millipolarisation units (mP) between the bound samples and the free tracer were plotted using GraphPadPrism. The dose-response curves were fitted to a one-site binding model. The

fraction bound (F) was calculated using the equations below, where r is the anisotropy, r_{max} is the maximum anisotropy, r_{min} is the minimum anisotropy, Int_{bound} is the fluorescence intensity in the bound state, and Int_{free} is the fluorescence intensity in the unbound state.[8]

$$F = \frac{r - r_{min}}{R\left(r_{max} - r\right) + r - r_{min}}$$

$$R = \frac{Int_{bound}}{Int_{free}}$$

Competition assays were used to determine the affinity of CODD and hyCODD for tPHD2 using the same buffer conditions and cofactor concentrations as above with the addition of 5 % (*v*/*v*) DMSO. A 2-fold dilution series of competitor peptides from 1000 μM to 15.6 μM were used with the following protein concentrations: 3 μM for *apo*-tPHD2, 1.5 μM for *apo*-tPHD2.Zn, 0.5 μM for *apo*-tPHD2.Zn.2OG. Data were fitted using a competitive model for the peptides with the top and bottom values of the fit constrained for each condition to be the same for experiments with CODD and hyCODD. The fluorescent tracer used (CODD*) was an *N*-terminally fluorescein isothiocyanate (FITC)-labelled HIF-1α CODD peptide (sequence: FITC-DLDLEMLAPYIPMDDDFQL; synthesised by GL Biochem (Shanghai) Ltd., China).

Isothermal Titration Calorimetry Experiments

Isothermal titration calorimetry (ITC) experiments were performed using a Malvern Microcal PEAQ-ITC automated machine. A solution of peptide (CODD or HyCODD, 500 μM) in ITC buffer (100 mM Tris-HCl pH 7.5 at 4 °C, 100 mM NaCl, 3 % DMSO (*v*/*v*)) was titrated using one injection of 0.4 μL followed by 18 injections of 2 μL into a solution of *apo*-tPHD2 (44 μM) in ITC buffer. Data were fitted to single-site binding model using MicroCal PEAQ-ITC analysis software v. 1.1.0.1262. For experiments with cofactors, the ITC buffer was supplemented with $ZnSO_4$ (1 mM) and 2OG (10 mM). Peptide concentrations were

established by ^{1}H NMR with an internal standard. In the experiments using tPHD2.Zn.2OG, to preclude the possibility of protein degradation giving a negative result, the same sample of protein used for both experiments and the titration using HyCODD was run before the titration with CODD.

NMR Experiments

Nuclear Magnetic Resonance (NMR) spectra were recorded using a Bruker AVIII 700 MHz NMR spectrometer equipped with a 5-mm inverse cryoprobe using 5 mm diameter NMR tubes (Norell) or 3 mm MATCH NMR tubes (Cortectnet). Data were processed with Bruker 3.1 software.

CPMG-edited ^{1}H NMR Experiments

Typical experimental parameters for ^{1}H Carr-Purcell-Meiboom-Gill (CPMG) NMR spectroscopy were as follows: total filter time, 40 ms; relaxation delay, 2 s; and number of transients, 264. The PROJECT-CPMG sequence as described by Aguilar *et al.* was applied ($90°_x$–[τ–$180°_y$–τ–$90°_y$–τ–$180°_y$–τ]$_n$–acq).[9, 10] Water suppression was achieved by pre-saturation.

2OG Displacement Monitoring by CPMG-edited ^{1}H NMR

Assay mixtures contained 10 µM 2OG, 10 µM *apo*-tPHD2, supplemented with 80 µM Zn(II) and 400 µM of peptidic substrate (if necessary) buffered in 50 mM Tris-D_{11}/HCl (pH 7.5) and 0.02 % NaN_3 (*w*/*v*) in 90 % H_2O and 10 % D_2O (*v*/*v*).

Binding of TCA Cycle Intermediates to tPHD2 by CPMG-edited ^{1}H NMR

Assay mixtures contained 10 µM *apo*-tPHD2, 80 µM Zn(II) buffered and increasing concentrations of TCA cycle intermediates (10-400 µM), in 50 mM Tris-D_{11}/HCl, pH 7.5, in 90 % H_2O and 10 % D_2O (*v*/*v*).

TCA Cycle Intermediates Displacement Monitoring by CPMG-edited ^{1}H NMR

Assay mixtures contained 10 μM succinate or fumarate, 10 μM *apo*-tPHD2, 80 μM Zn(II) and 400 μM hyCODD (if necessary) buffered in 50 mM Tris-D_{11}/HCl, pH 7.5, in 90 % H_2O and 10 % D_2O (*v*/*v*).

^{1}H-^{15}N HSQC Experiments

For ^{1}H-^{15}N HSQC experiments, the Bruker pulse sequence *hsqcetfpf3gpsi* was used. Typically, the size of the FID for the ^{1}H dimension was 2048 points, and for the ^{15}N dimension was set as 128, 256, or 1024 points depending on the resolution required. The spectral width was set as 16 ppm (^{1}H) and 40 ppm (^{15}N) respectively, and the centre of the spectrum was set to 4.7 ppm and 120 ppm. $^1J_{NH}$ was set to 90 Hz and ^{15}N decoupling was achieved using the GARP sequence. The relaxation delay was 1 second.[11] Buffering was with 50 mM Tris-D_{11}/HCl, pH 6.6, in 95% H_2O, 5% D_2O (*v*/*v*).

^{13}C-2OG and ^{13}C-NODD/CODD Displacement Experiments

The CLIP-HSQC sequence was used for 1D ^{13}C HSQC experiments (without ^{13}C decoupling).[12] The relaxation delay was 2 s and the $^1J_{CH}$ was set to 160 Hz. A 6.8 ms Q3 180-degree pulse was used for selective ^{13}C irradiation at the chosen chemical shift. 2OG was 1,2,3,4-[^{13}C]-labelled and CODD/NODD was uniformly [^{13}C]-labelled on its prolyl ring. Assay mixtures contained 50 μM 2OG, 50 μM *apo*-tPHD2, 80 μM Zn(II), 50 μM NODD/CODD and 400 μM hyCODD (if necessary) buffered in 50 mM Tris-D_{11}/HCl, pH 7.5, in 90 % H_2O and 10 % D_2O (*v*/*v*).

2OG Turnover Monitoring by ^{1}H NMR

^{1}H NMR spectra were obtained at 150 s intervals and integrated using absolute intensity scaling to monitor changes in the intensity of signals of interest. The reaction was carried out at 310 K in a 5 mm diameter NMR tube (Norell), and initiated by addition of 2OG. HyCODD was added in around a 5-fold excess to the K_m of the peptidic substrate and

pre-incubated for 15 min[13] in a reaction mixture containing 1 μM *apo*-tPHD2, 50 μM Fe(II), 50 μM 2OG, 50 μM peptidic substrate (CODD/NODD) and 500 μM L-ascorbate in 50 mM Tris-D_{11}, pH 7.5.

^{19}F NMR Monitoring of Labelled Peptides

^{19}F NMR experiments were recorded using a Bruker AVIII 600 MHz NMR spectrometer equipped with a broad band BB-^{19}F/^{1}H Prodigy N_2 cryoprobe using 5 mm diameter NMR tubes (Norell). Spectra were typically obtained using 2000 scans and a recovery delay of 2 s. Assay mixtures contained 20 μM tPHD2, 50 μM Fe(II), 500 μM 2OG, 1 mM L-ascorbate, and 400 μM DLDLEMLAP*YIPMDDDFQL-NH_2 (*C4 proline ^{19}F-labelled *cis* or *trans*) buffered in 50 mM Tris-HCl, pH 7.5, in 90 % H_2O and 10 % D_2O (*v*/*v*).

Docking

Models for tPHD2.Mn.succinate.HIF-1αCODD$_{558\text{-}574}$ (C4-*endo* P564), tPHD2.Mn.succinate.HIF-1αCODD$_{558\text{-}574}$ (C4-*exo* Hyp564), and tPHD2.Mn.succinate.HIF-1αCODD$_{558\text{-}574}$ (C4-*endo* Hyp564) were generated using crystal structures of tPHD2.Mn.2OG.HIF-1αCODD$_{558\text{-}574}$ (C4-*endo* P564) (PDB: 5L9B),[11] VCB.HIF-1αCODD$_{558\text{-}574}$ (C4-*exo* Hyp564) (PDB: 1LM8),[14] and a modelled collagen triple helix (C4-*endo* Hyp) (PDB: 3B2C)[15] complexes as templates using COOT.[16] Models for tPHD2.Mn.2OG.HIF-1αCODD$_{558\text{-}574}$ (C4-*exo* Hyp564) and tPHD2.Mn.2OG.HIF-1αCODD$_{558\text{-}574}$ (C4-*endo* Hyp564) were generated using crystal structures of VCB.HIF-1αCODD$_{558\text{-}574}$ (C4-*exo* Hyp564) (PDB: 1LM8)[14] and a modelled collagen triple helix (C4-*endo* Hyp) (PDB: 3B2C)[15] complexes as templates using COOT.[16] All models were conjugate energy minimized to ensure their geometric quality without applying any external energy term.

Computational Studies

MD Simulation Studies

Two structures of tPHD2.Mn.CODD were studied; one with Hyp564 in the C4 *cis* conformation and one with Hyp564 in the C4 *trans* conformation. For each conformation, two different co-substrate/product binding modes were considered, *i.e.* bidentate binding with 2-oxoglutarate (2OG) and monodentate binding with succinate. In total, four systems were prepared, as indicated in Table S1. The protein was solvated using the Solvate plug-in of VMD,[17] resulting in a box size of (82x82x82) $Å^3$. The system was ionised to be electrically neutral using a 0.15 mM background ionic concentration of NaCl. Protonation states were chosen corresponding to pH 7.0, with δ-His313 and δ-His374 protonation states in the active site.

For each system, we first tested the validity of the initial PDB derived model of tPHD2, using a substrate conformation obtained based on semi-automated (knowledge-based) docking. The initial MD simulation system setup was based on the structure of tPHD2.Mn.CODD.2OG[11] with Mn(II) exchanged for Fe(II). The MD simulation (10 ns) was performed for the initial modelled complex with backbone restraints of tPHD2 chain A, as well as the active site residues H313, D315, H374, 2OG or succinate, Fe(II) replacing the Mn(II) density, and H564 from the substrate peptide. Following this, MD production runs in the NPT ensemble were performed for 40 ns for each system, totalling 200 ns, keeping only the active site restrained, using a system temperature of 300 K and 1 bar pressure.

The protein conformation was simulated using the CHARMM 36 force-field (FF), with the CMAP correction;[18] a TIP3P water model was employed.[19] CHARMM FF parameters for the 2OG ligand compatible with version 2b7 of the CHARMM General Force-Field (CGenFF)[20-23] were generated and validated using the Force-Field toolkit plugin in

VMD,[24] after initial atom typing and assignment of parameters and charges using version 0.9.7 of the CGenFF program at paramchem.org.[21, 23]

QM/MM Studies

Using the MD simulation system setups, we performed quantum mechanics/molecular mechanics (QM/MM) geometry optimisation using the CP2K code[25] (http://cp2k.berlios.de). Details of the systems studied are summarized in Table S1. The interaction energy for the QM region was computed via the QuickStep module within CP2K,[26, 27] with the FIST MM driver, and using a real-space multigrid technique for the electrostatic coupling between the QM and MM regions.[28, 29] The QM region was treated using density functional theory (DFT) with the BLYP exchange correlation energy functional[30, 31] employing the GTH pseudopotential of Goedecker *et al.*,[32, 33] and double-zeta valence plus polarisation (DZVP) basis sets (optimised for BLYP) as implemented in CP2K. The Fe(II) metallocenter was described using the DZVP basis sets optimised for BLYP. The plane wave was expanded up to a density cut-off of 300 Ry. For the MM region, the CHARMM 36 force-field49 was used with the TIP3P water model, via the FIST module within CP2K to calculate the MM interaction energy. Interactions between the QM and MM regions were calculated using the procedure of Laino *et al.*[28] MM and QM optimisations were performed separately, with the QM/MM interface described with the IMOMM link-atom method,[34] where positions of hydrogen capping atoms were expressed as a function of the coordinates of atoms forming the original bond, and the forces on the link atoms were accordingly redistributed. A scaling factor of 1.38 was applied to relate the MM carbon–carbon distances to the QM carbon–hydrogen ones. Only succinate/2OG, D315, Fe(II), and Hyp564 was included in the QM region; the rest of the protein was treated at the MM level. The total charge of the QM region in each system is summarised in Table S2. Geometry optimisations were performed up to 200 iterations. For every step, the electronic structure was explicitly quenched to a tolerance of

10^{-14} hartree, and a convergence criteria of 10^{-3} for the RMS force and gradient was employed.

Supplementary Figures

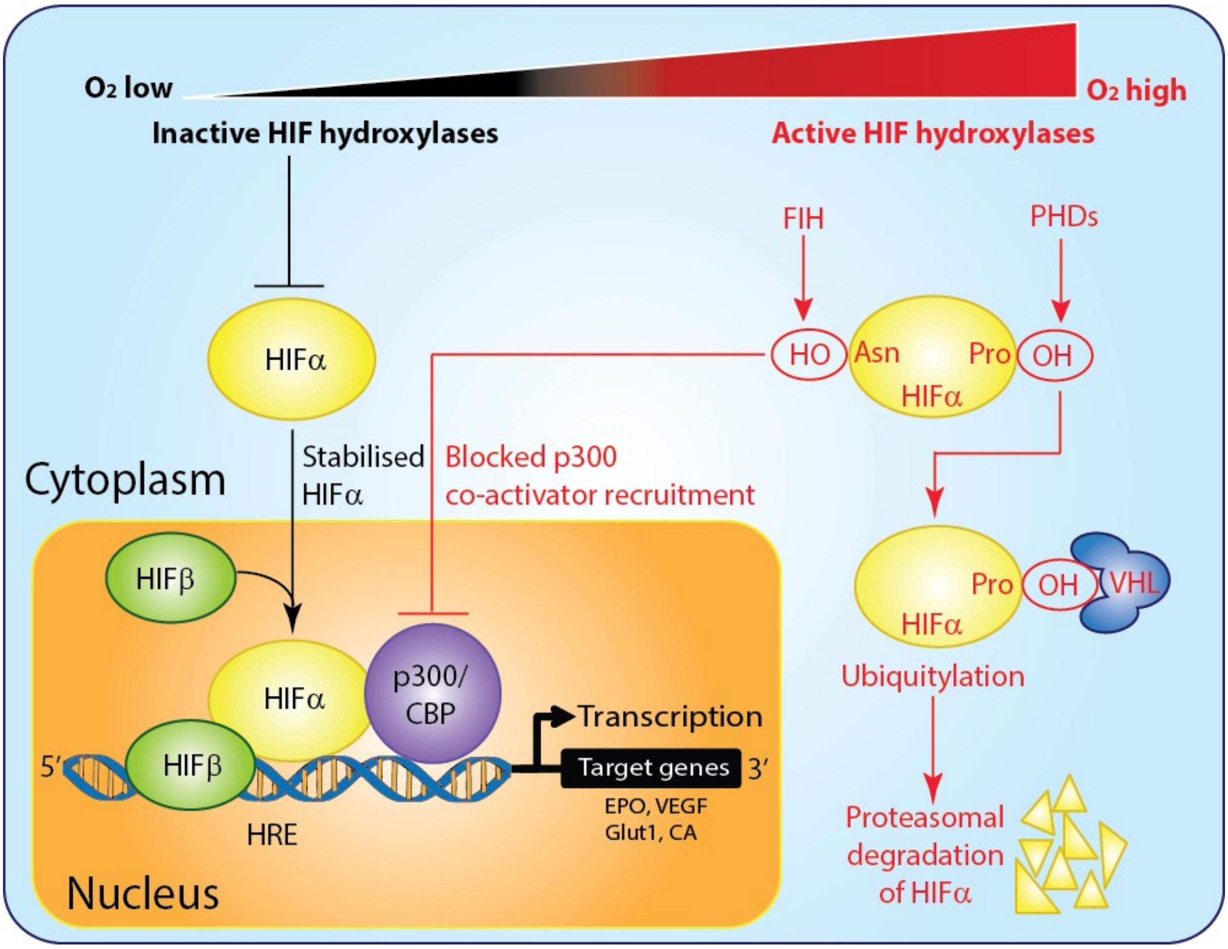

Fig. S1. Outline of the machinery involved in the cellular response to hypoxia in animals. *A simplified scheme depicting the cellular response to hypoxia and normoxia in animals. PHD: prolyl hydroxylase domain, HIF: hypoxia inducible factor, FIH: factor inhibiting HIF, VHL: von Hippel-Lindau protein, CBP: CREB binding protein, HRE: hypoxia response element, EPO: erythropoietin, VEGF: vascular endothelial growth factor, Glut1: glucose transporter, CA: carbonic anhydrase.*

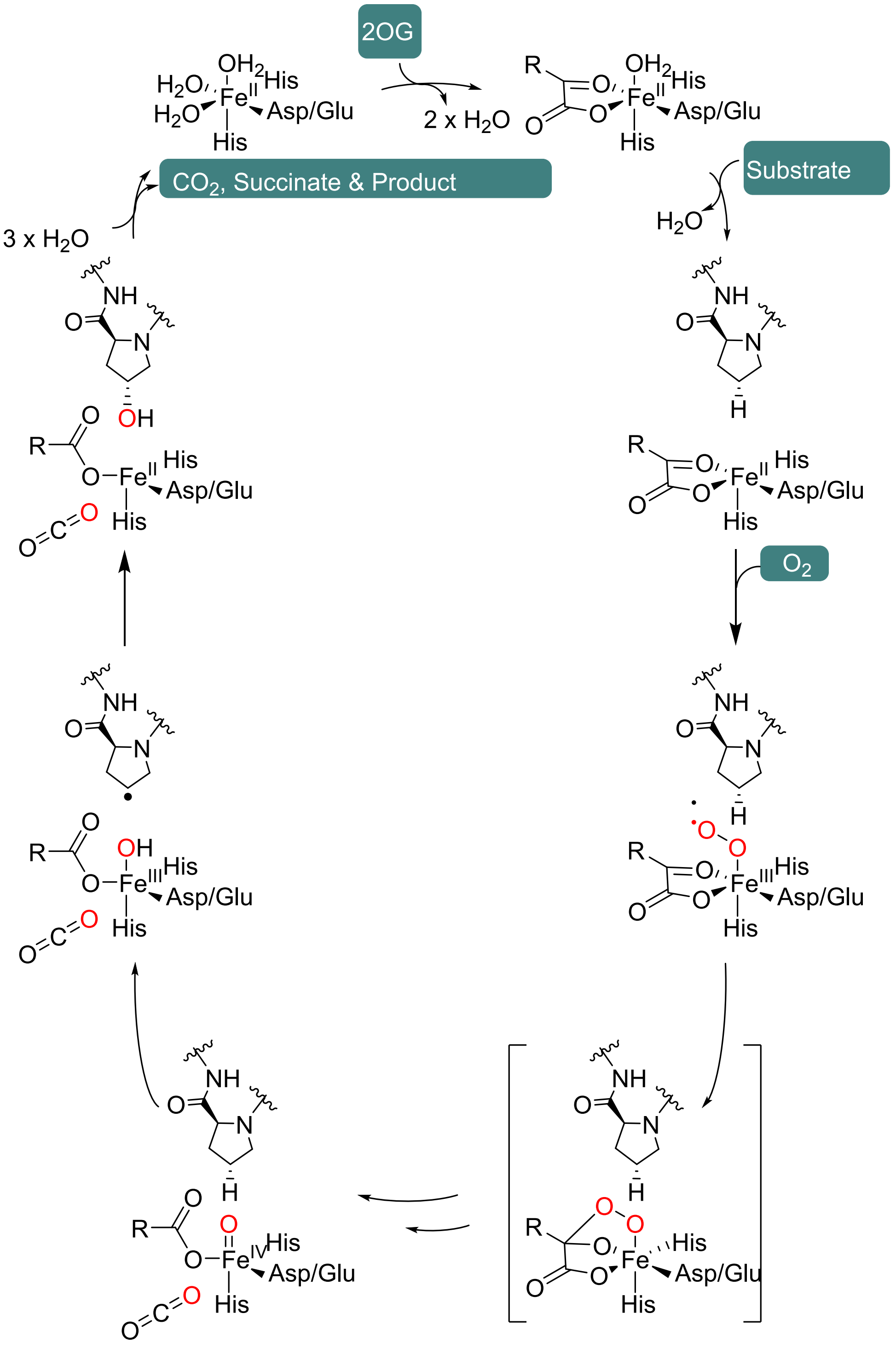

Fig. S2. Outline mechanism for PHD2 catalysed HIF-α hydroxylation.[35]

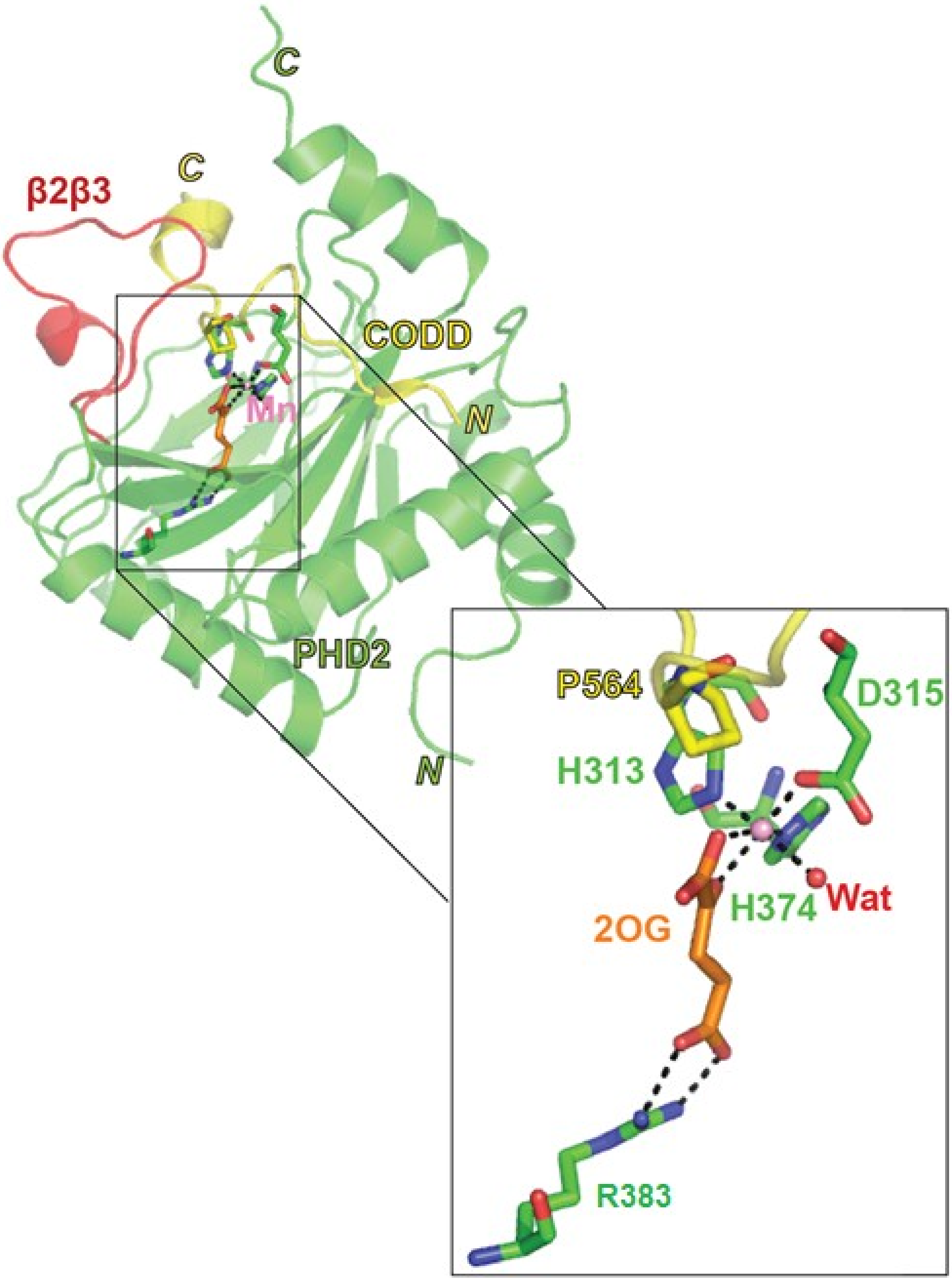

Fig. S3. View from a crystal structure of tPHD2 in complex with metal, 2-oxoglutarate (2OG), and CODD (PDB ID: 5L9B).[11] *The tPHD2 residues 237-254, which adopt a finger-like β2β3 conformation in the tPHD2 crystal form (PDB ID: 2G1M),[4] fold to adopt a loop conformation in the tPHD2.CODD complex structure, enclosing the Pro-564$_{HIF\text{-}1\alpha}$ region and the tPHD2 active site. 2OG coordinates the active site metal via one of its carboxylate oxygens and its amide α-carbonyl oxygen; its binding is stabilised by ionic interactions between its carboxylate and Arg-383$_{tPHD2}$.*

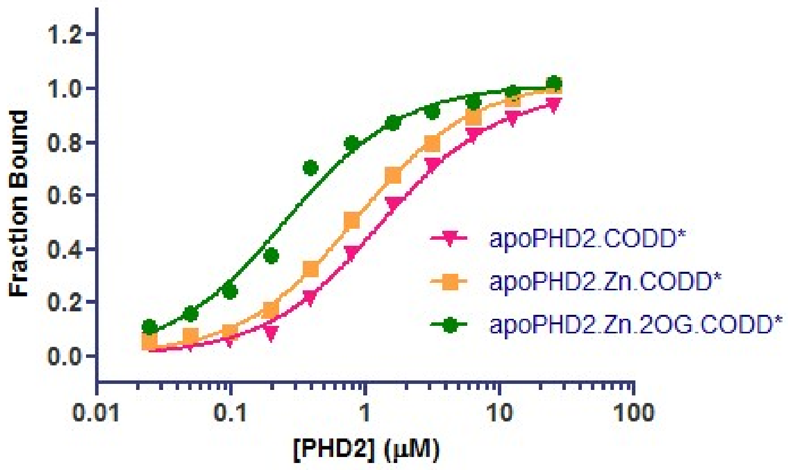

Fig. S4. Fluorescence polarisation studies of the binding of CODD* to tPHD2. *A dose response experiment was first performed to determine the dissociation constant (K_D) for tPHD2 binding of the fluorescent tracer (i.e. N-terminally fluorescein isothiocyanate (FITC)-labelled HIF-1α CODD peptide; CODD*). After determining the best conditions for the tracer, competitive binding assays were conducted by titrating increasing concentrations of competing ligands to a fixed (optimal) tracer and tPHD2 concentrations (Fig. S5). The assay conditions are given in the Materials and Methods.*

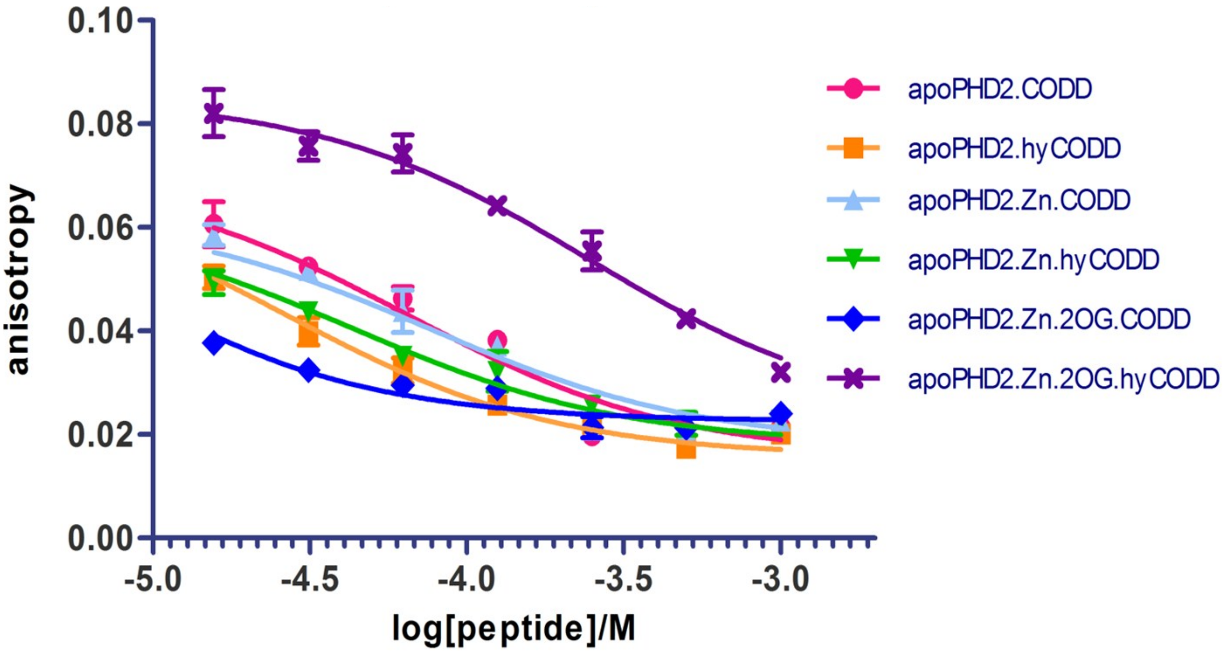

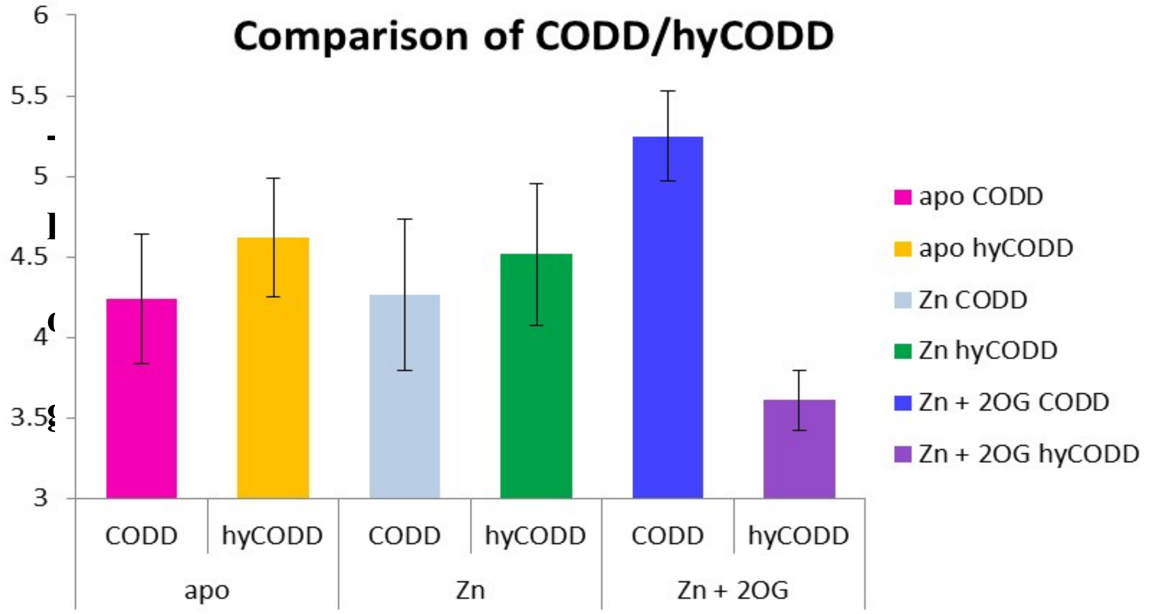

Fig. S5. Fluorescence polarisation studies on the binding of CODD and hyCODD to tPHD2. ***(A)*** *Curves and* ***(B)*** *bar charts showing the changes in CODD and hyCODD binding to tPHD2 under various conditions as observed by fluorescence polarisation (n = 3) using a Pherastar plate reader. Error bars represent standard deviation. The assay conditions are given in the Materials and Methods.*

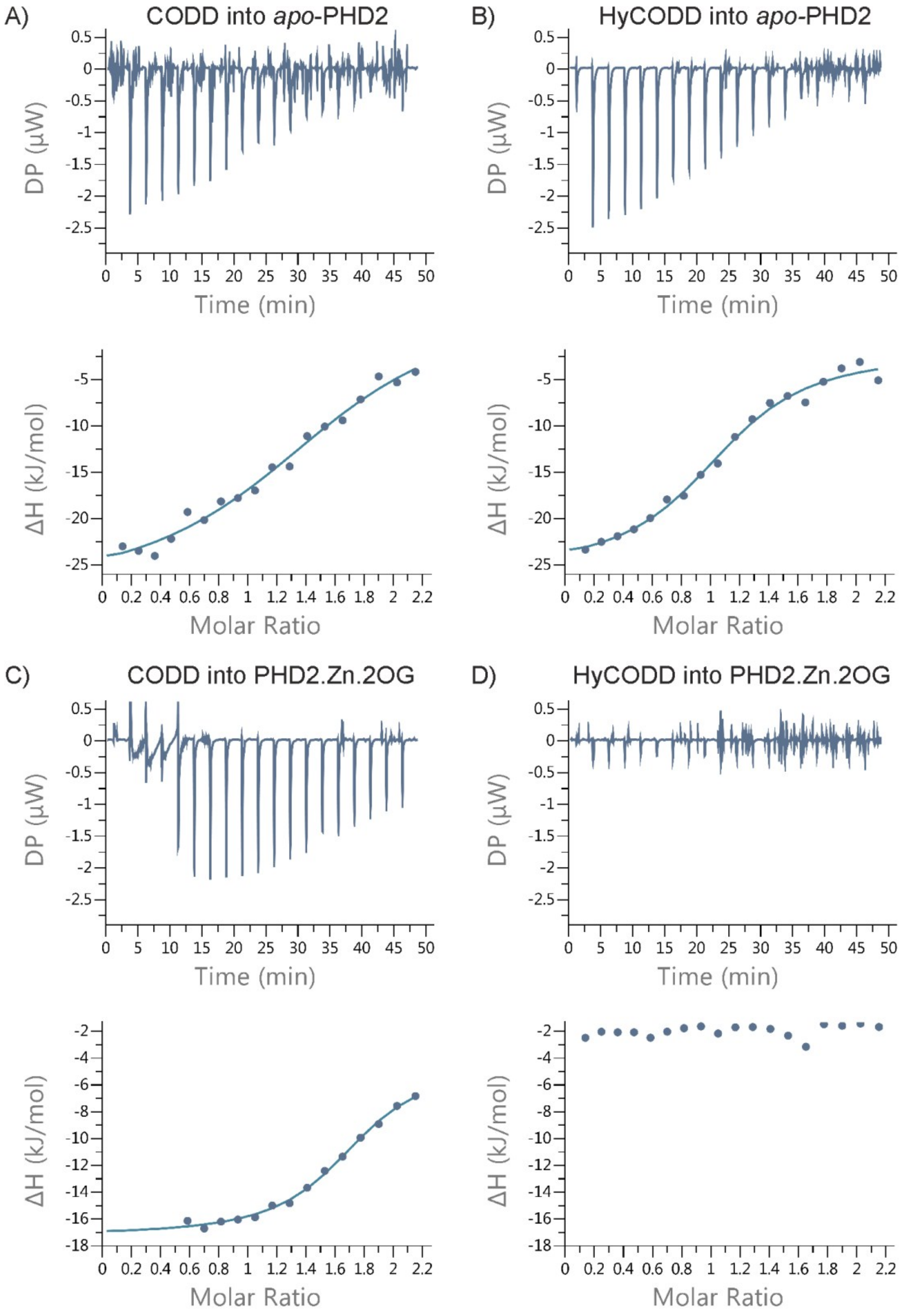

Fig. S6. Isothermal titration calorimetry (ITC) studies on the binding of CODD and hyCODD to (A) *apo*-tPHD2 and (B) tPHD2.Zn.2OG. *The K_D values were similar for CODD and hyCODD with apo-tPHD2 (9.41 ± 4.48 µM and 4.62 ± 1.36 µM, respectively). CODD was observed to bind to tPHD2.Zn.2OG (K_D = 1.78 ± 0.37 µM); however, no binding was observed for hyCODD to tPHD2.Zn.2OG, within detection limits (n= 3). Assay conditions are given in the Materials and Methods.*

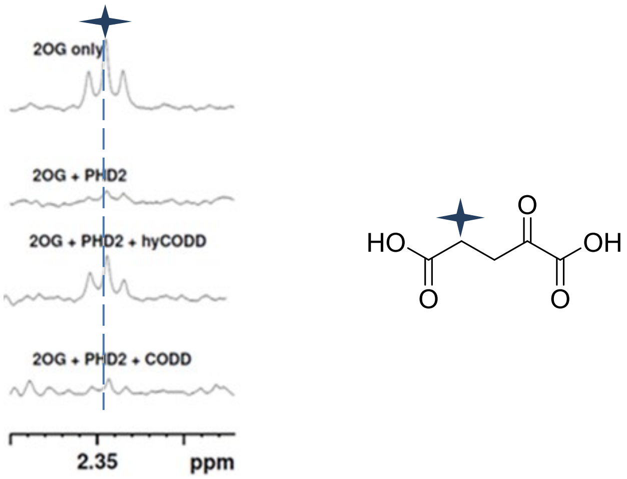

Fig. S7. Investigations of the binding mode of hyCODD to tPHD2.Zn using CPMG-edited ^{1}H NMR 2OG displacement analyses. *Assay mixtures contained 10 μM 2OG; 10 μM apo-tPHD2, 80 μM Zn(II) and 400 μM CODD/hyCODD (where necessary), buffered in 50 mM Tris-D_{11}/HCl, pH 7.5, in 90 % H_2O and 10 % D_2O (v/v). The resonance observed is indicated by a black star.*

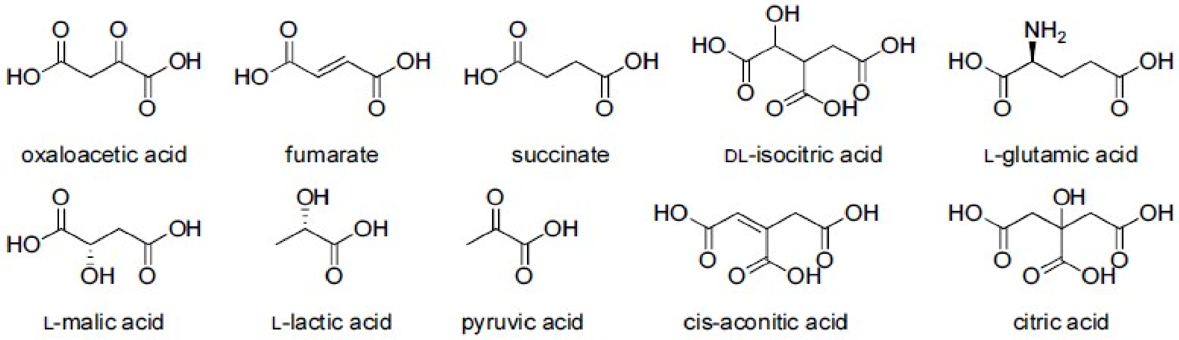

Fig. S8. Structures of the TCA cycle intermediates tested for binding to tPHD2.Zn. *Assay mixtures contained 10-400 μM TCA cycle intermediate, 10 μM apo-tPHD2, 80 μM Zn(II) buffered in 50 mM Tris-D_{11}/HCl, pH 7.5, in 90 % H_2O and 10 % D_2O (v/v). Only fumarate and succinate were observed to weakly bind to tPHD2 by CPM-edited ^{1}H NMR analyses.*

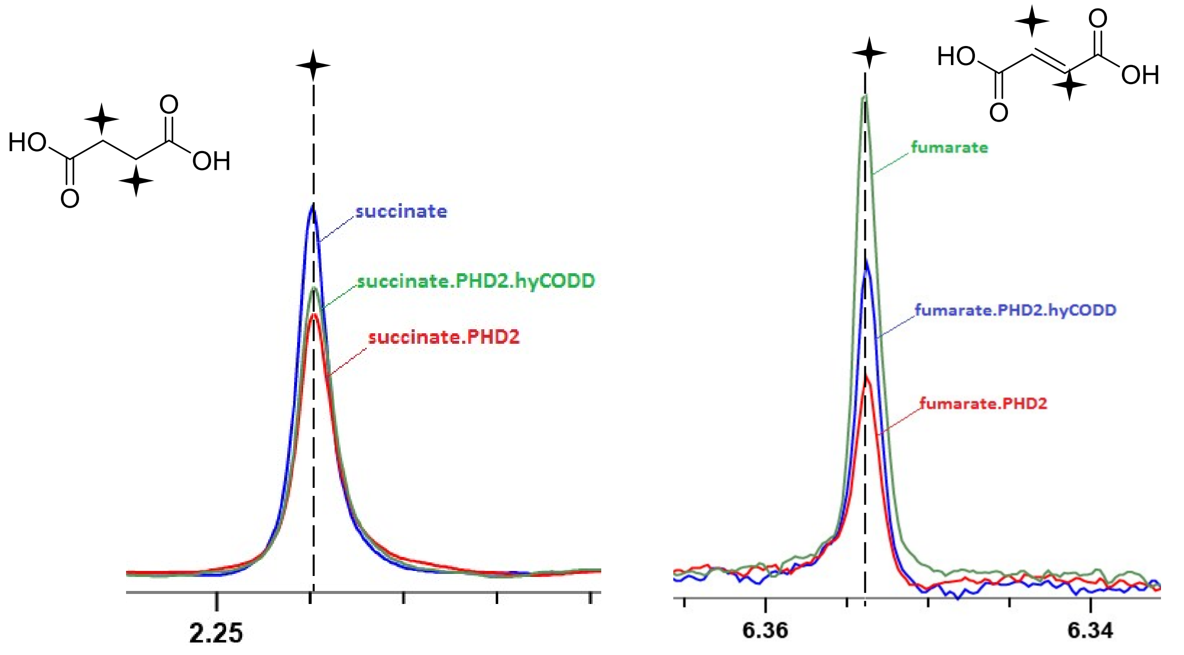

Fig. S9. Investigations of the binding of TCA cycle intermediates to tPHD2.Zn in the presence of Zn(II) and hyCODD by CPMG-edited ^{1}H NMR analyses. *Assay mixtures contained 10 µM succinate/fumarate (blue), 10 µM apo-tPHD2 (red), 80 µM Zn(II) and 400 µM hyCODD (green) buffered in 50 mM Tris-D_{11}/HCl, pH 7.5, in 90 % H_2O and 10 % D_2O (v/v). The resonances observed are indicated by black stars.*

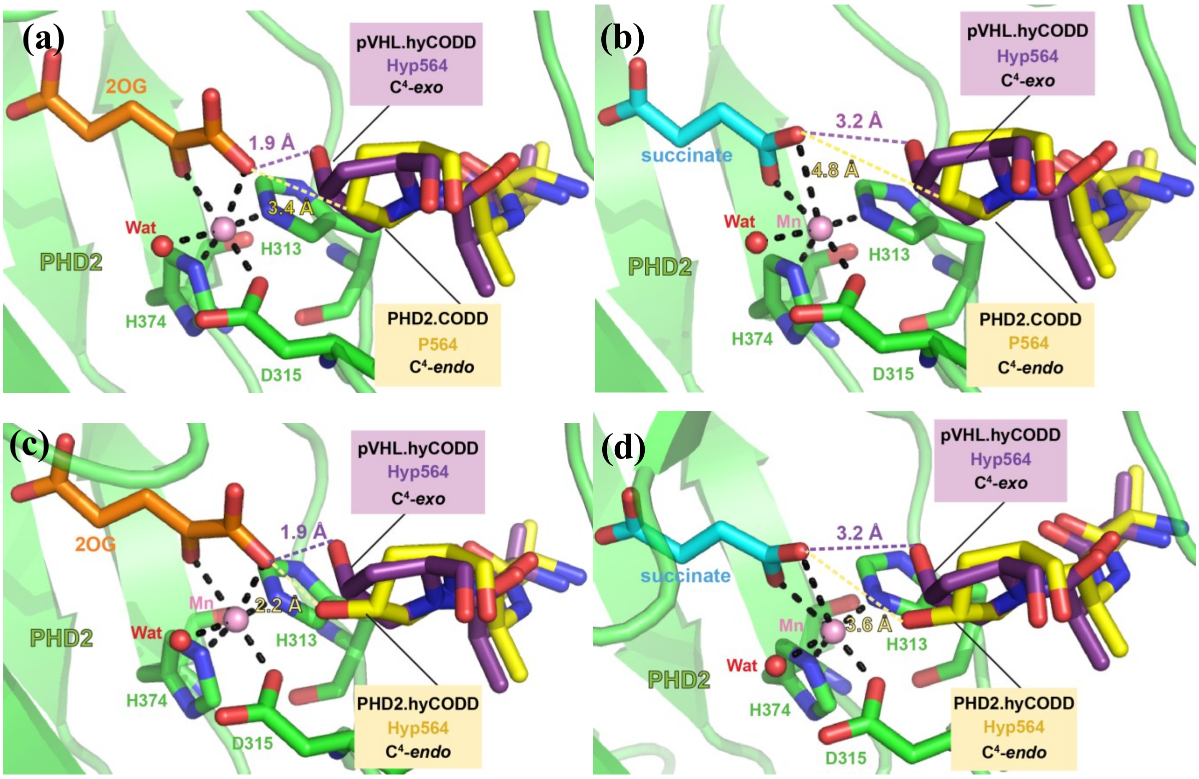

Fig. S10. Views derived from tPHD2 crystal structures evaluating potential clashes between metal complexed 2OG or succinate carboxylate and the prolyl alcohol of hyCODD. *(a) Views from a structure of tPHD2.Mn.2OG.CODD complex (PDB ID: 5L9B)*[11] *overlaid with that of hyCODD bound to pVHL (PDB ID: 1LQB.)*[36] *Note the proximity of the 2OG C1 carboxylate oxygen and the 'modelled' C4 alcohol (1.9 Å), assuming the prolyl residue adopts the C4-exo conformation as observed in the pVHL complex. In the case of the crystallographically observed C4-endo product complex in the tPHD2, the analogous distance is substantially longer (3.4 Å). (b) When succinate is modelled in the tPHD2 active site (using PDB ID: 5L9B)*[11] *the analogous distances are 3.2 Å and 4.8 Å for the C4-exo or C4-endo conformation, respectively. (c) This figure is the same as S9a, but with a 'trans' hydroxyl group manually added C4 of P564. Note the greater potential for a steric clash with the C4-exo (1.9 Å) compared to the C4-endo (2.2 Å) conformation (as previously proposed).*[37] *(d) The figure is the same as S9b, but with a 'trans' hydroxyl group manually added to C4 of P564. Note the analysis suggested both the C4-endo and exo of hyCODD will be accommodated equally well. tPHD2 is in green, CODD in yellow, hyCODD in purple, succinate in cyan, and 2OG in orange. The figure was created with PyMOL. The overall analyses imply the potential for a clash is the greatest with 2OG and the C4-exo hyCODD prolyl conformation, consistent with the experimental results (Fig. 2 and S6).*

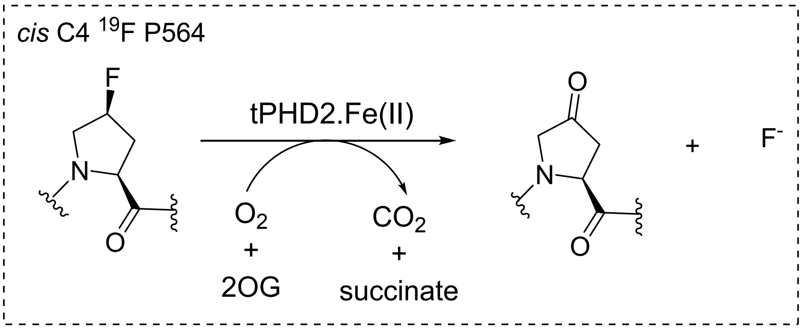

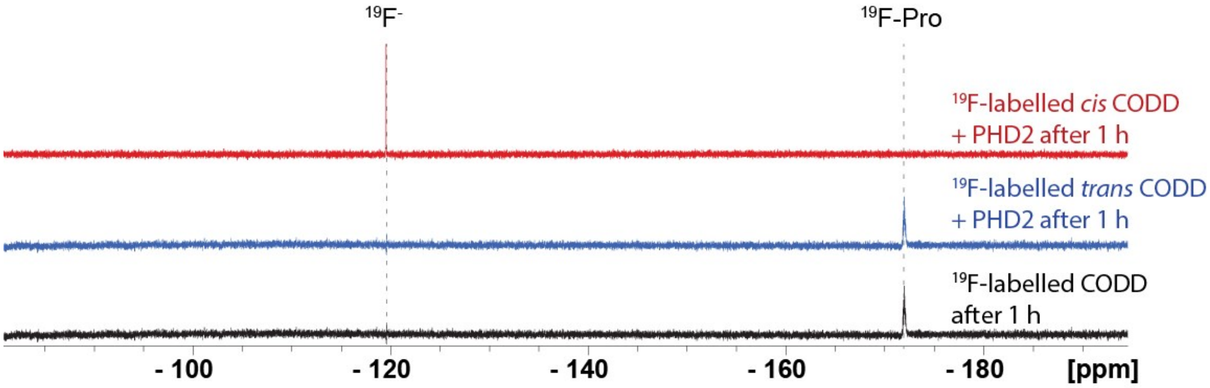

Fig. S11. ^{19}F NMR monitoring of fluoride release by tPHD2 using ^{19}F-labelled *cis* or *trans* P564 CODD as a substrate. *Assay mixtures contained 20 µM tPHD2, 50 µM Fe(II), 500 µM 2OG, 1 mM ascorbate, and 400 µM cis or trans C4 ^{19}F P564 CODD*[37] *buffered in 50 mM Tris-HCl, pH 7.5, in 90 % H_2O and 10 % D_2O (v/v). Red trace: after 1 h. Fluoride release (-120 ppm) was detected in the presence of tPHD2.Fe.2OG with cis C4-fluoro-CODD and was confirmed by spiking the samples with sodium fluoride. The peak corresponding to the ^{19}F-Pro is at -172 ppm. No evidence for substantial binding of trans C4 ^{19}F P564 CODD to tPHD2.Fe.2OG (blue trace) was observed, as indicated by the lack of significant intensity changes of the fluorinated peptide peak at -172 ppm compared to the black trace (in the absence of protein), consistent with predicted binding of the C4-endo prolyl conformation.*[37] *Spectra were referenced to the internal standard trifluoroacetic acid (TFA) at -75.2 ppm.*

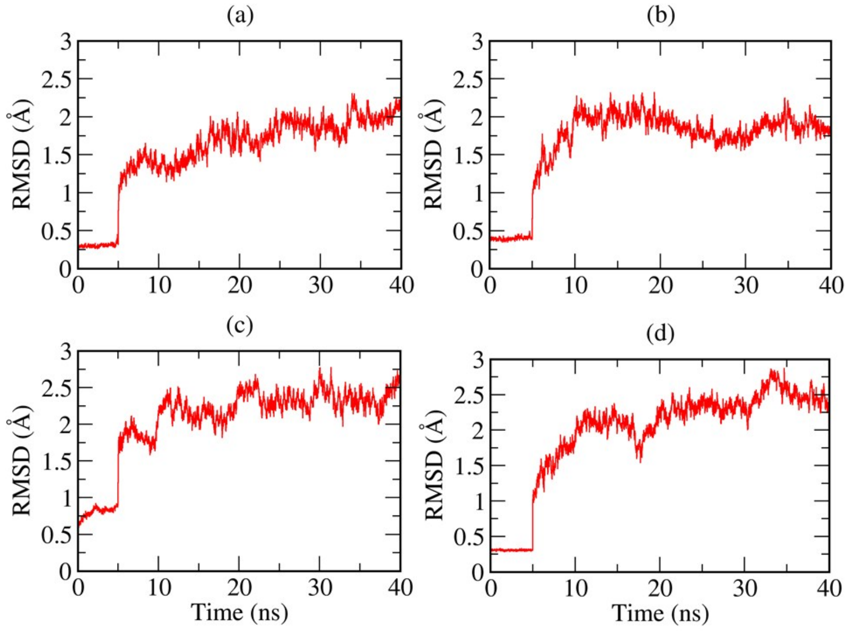

Fig. S12. The observed tPHD2 backbone RMSD (Å) in MD simulations of tPHD2 complexes. *(a) tPHD2.Fe.succinate.CODD cis-Hyp564, (b) tPHD2.Fe.2OG.CODD cis-Hyp564, (c) tPHD2.Fe.succinate.CODD trans-Hyp564, and (d) tPHD2.Fe.2OG.CODD trans-Hyp564.*

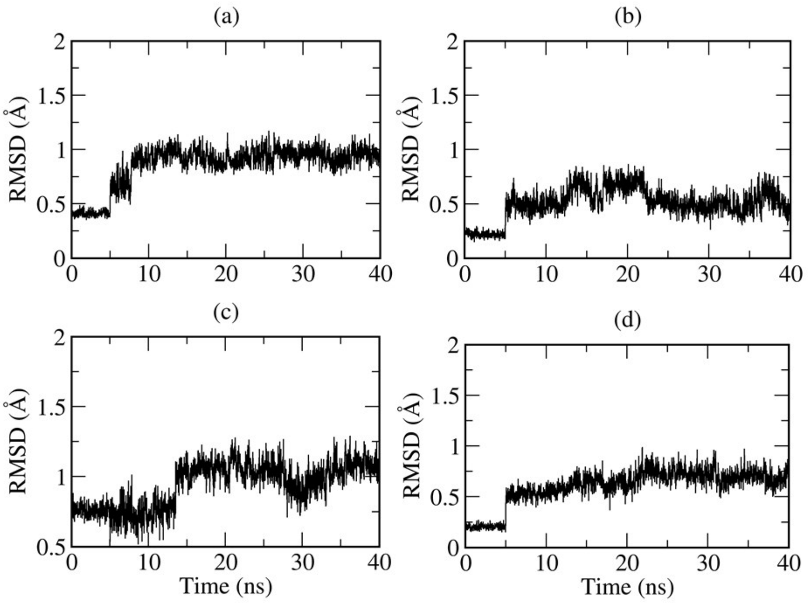

Fig. S13. The observed active site RMSD (Å) in MD simulations of tPHD2 complexes. *(a) tPHD2.Fe.succinate.CODD cis-Hyp564, (b) tPHD2.Fe.2OG.CODD cis-Hyp564, (c) tPHD2.Fe.succinate.CODD trans-Hyp564, and (d) tPHD2.Fe.2OG.CODD trans-Hyp564. The active site is defined as consisting of residues F366, H374, D315, H313, Hyp564, Tyr303 as well as succinate or 2-oxoglutarate (2OG).*

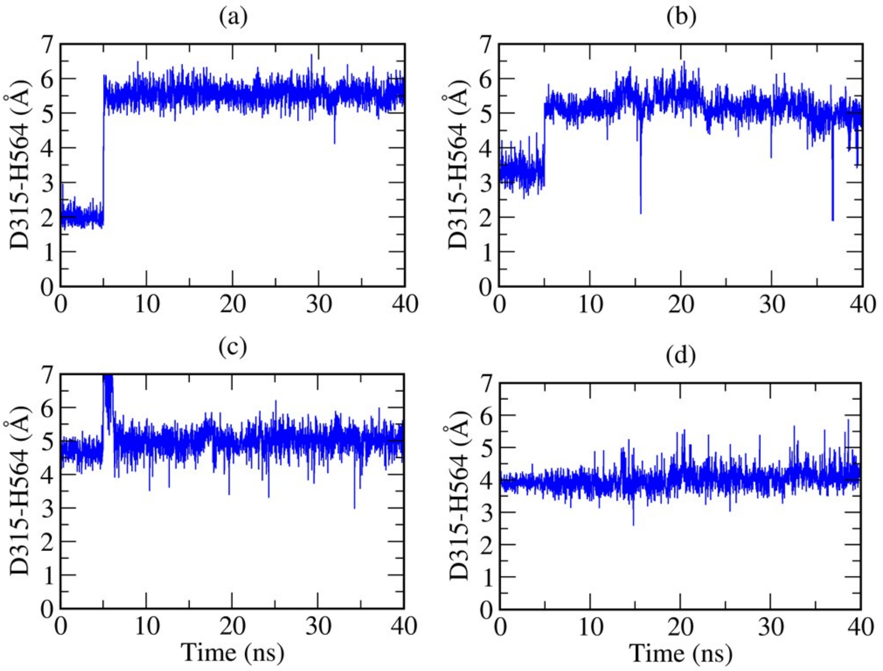

Fig. S14. The observed D315-H564 distance ($O_{\delta 1}$-Hε) (Å) in MD simulations of tPHD2 complexes. *(a) tPHD2.Fe.succinate.CODD cis-Hyp564, (b) tPHD2.Fe.2OG.CODD cis-Hyp564, (c) tPHD2.Fe.succinate.CODD trans-Hyp564, and (d) tPHD2.Fe.2OG.CODD trans-Hyp564.*

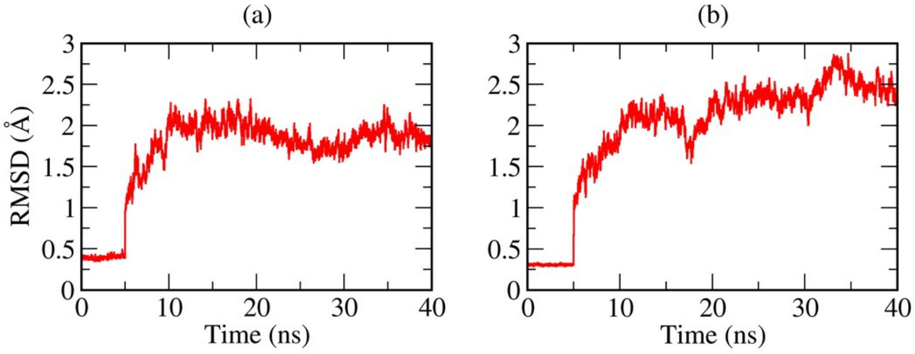

Fig. S15. The observed backbone RMSD (Å) using MD simulations of tPHD2.Fe.2OG complexed with (a) CODD *cis*-Hyp564 or (b) CODD *trans*-Hyp564.

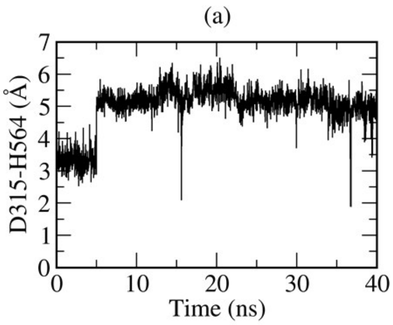

Fig. S16. The observed tPHD2 backbone RMSD (Å) using simulations of tPHD2.Fe complexed with (a) CODD *cis*-Hyp564 or (b) CODD *trans*-Hyp564.

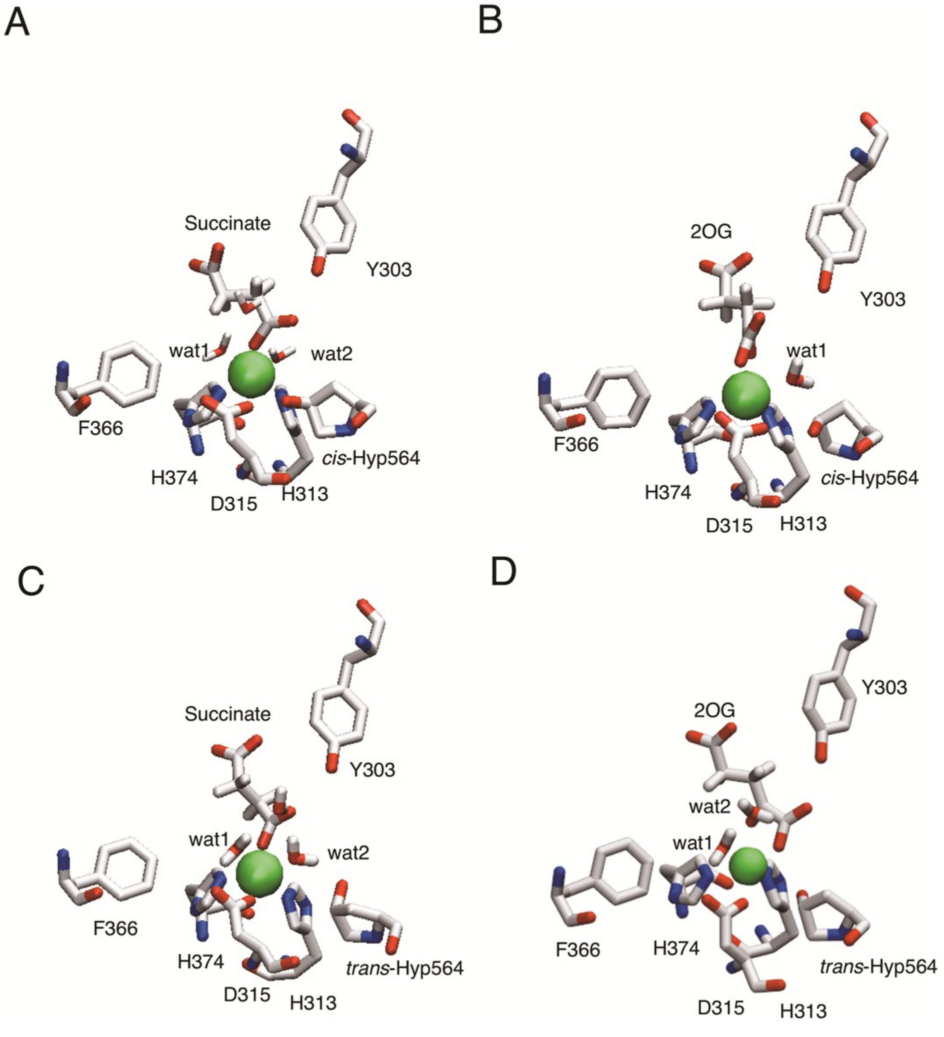

Fig. S17. Views of the tPHD2 active site as observed in snapshots at the RMSD plateau from the classical MD production runs. *(A) tPHD2.Fe.succinate.CODD cis-Hyp564, (B) tPHD2.Fe.2OG.CODD cis-Hyp564, (C) tPHD2.Fe.succinate.CODD trans-Hyp564, and (D) tPHD2.Fe.2OG.CODD trans-Hyp564.*

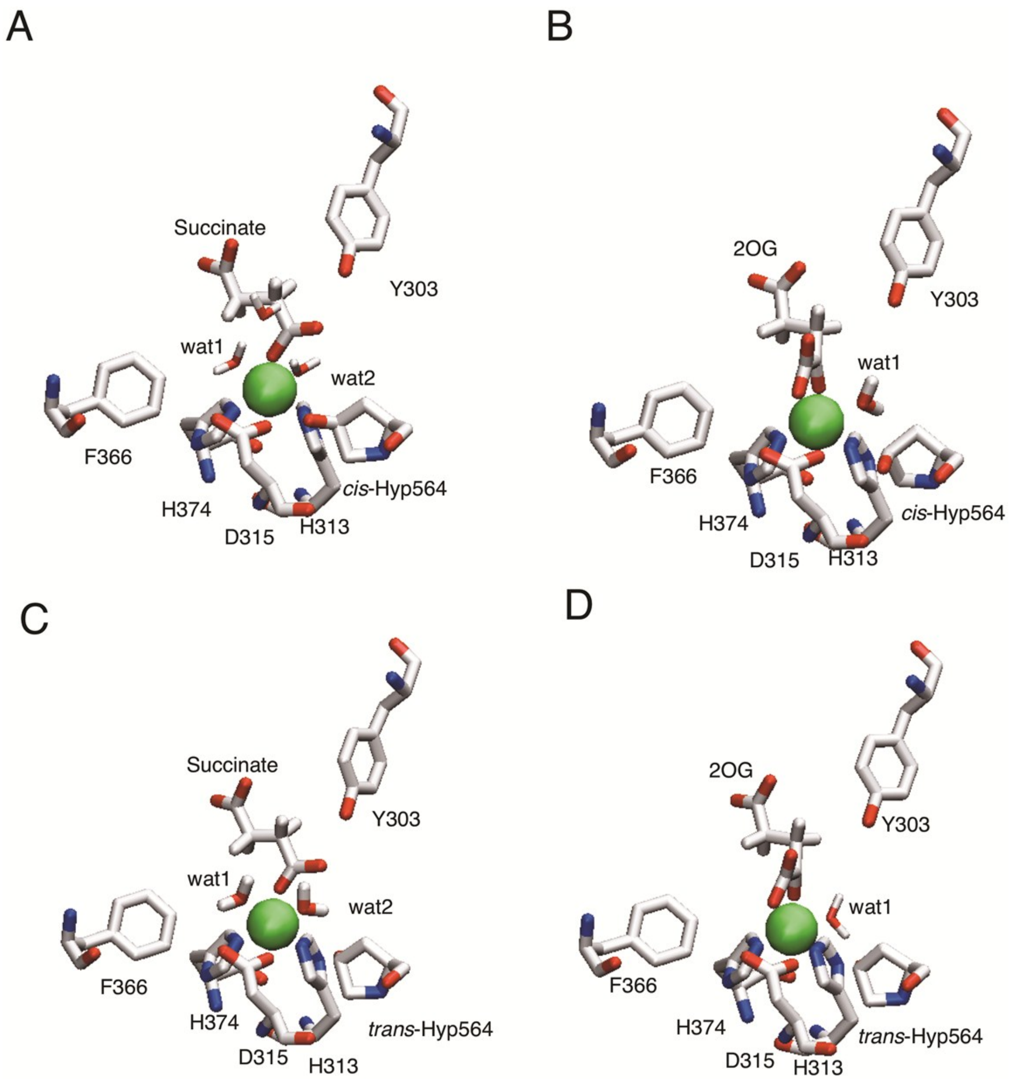

Fig. S18. Views of the tPHD2 active site as observed in QM/MM minimised snapshots. *(A) tPHD2.Fe.succinate.CODD cis-Hyp564, (B) tPHD2.Fe.2OG.CODD cis-Hyp564, (C) tPHD2.Fe.succinate.CODD trans-Hyp564, and (D) tPHD2.Fe.2OG.CODD trans-Hyp564.*

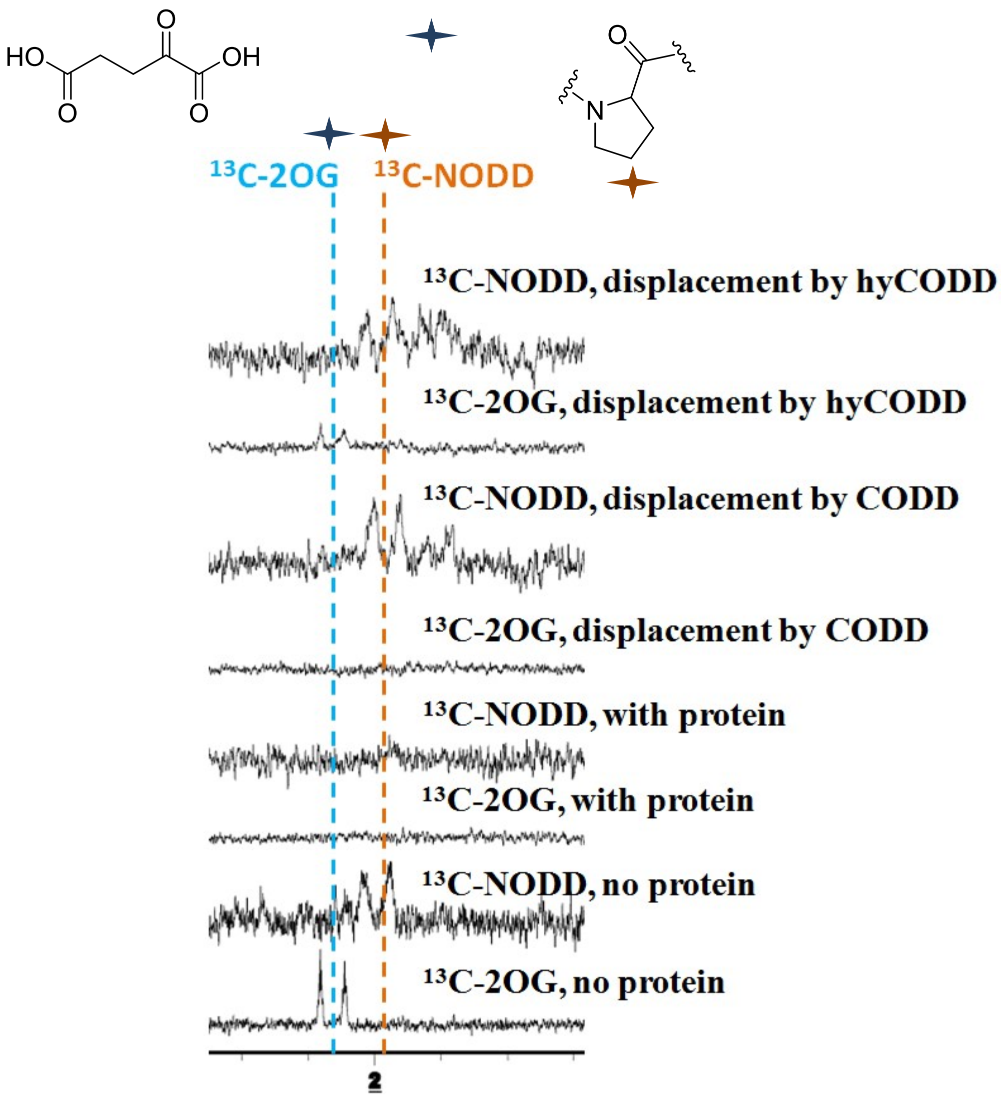

Fig. S19. 1D CLIP-HSQC (with selective ^{13}C-inversion) analyses of the binding of hyCODD to the tPHD2.Zn.2OG.NODD complex. *Assay mixtures contained 50 µM 2OG, 50 µM apo-tPHD2, 80 µM Zn(II), 50 µM NODD and 400 µM hyCODD (if necessary) buffered in 50 mM Tris-D_{11}/HCl, pH 7.5, in 90 % H_2O and 10 % D_2O (v/v). ^{13}C-2OG was labelled at carbon positions 1, 2, 3 and 4, and ^{13}C-NODD was uniformly labelled at all carbon atoms of its proline ring. ^{13}C-2OG/^{13}C-NODD selective excitation positions are indicated by coloured stars. NMR assignments have been previously reported.*[11]

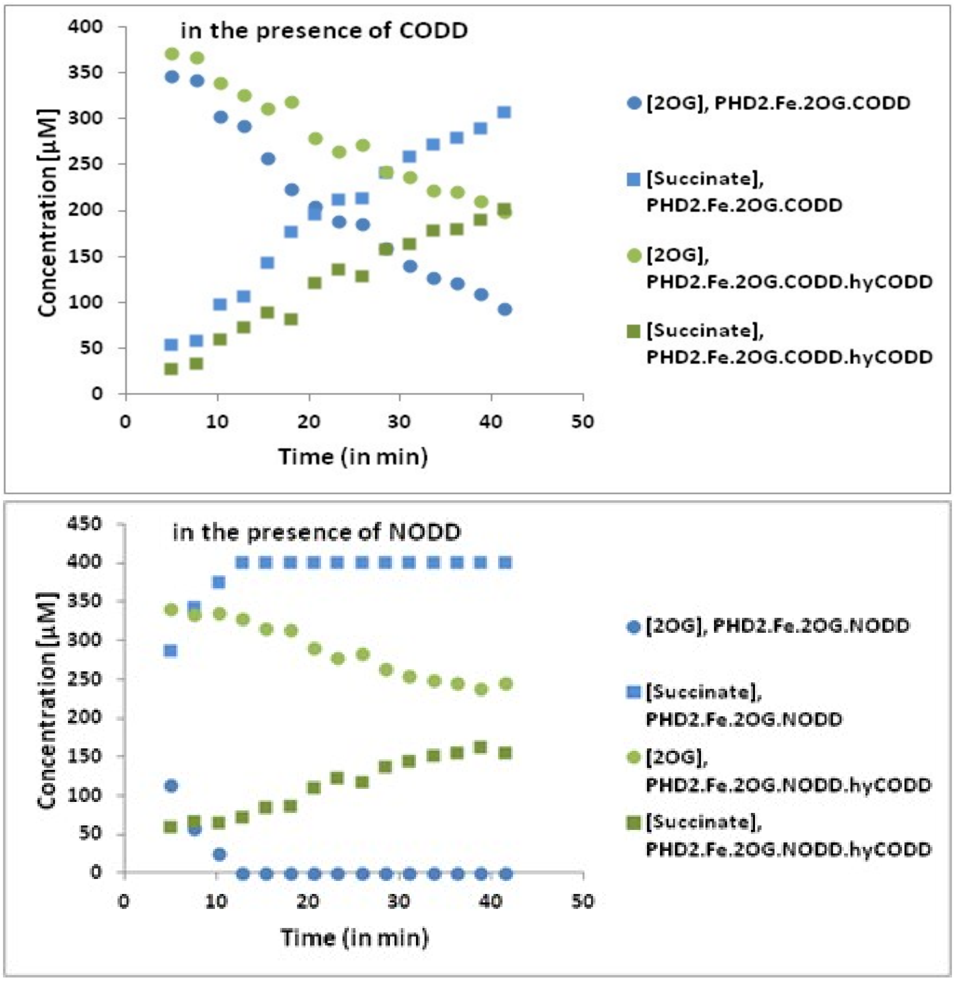

Fig. S20. Monitoring of tPHD2 catalysed 2OG to succinate turnover in the presence of added hyCODD as observed by ^{1}H NMR using either CODD or NODD as a substrate. *Assay mixtures contained 50 mM Tris-D_{11}/HCl, pH 7.5, in 90 % H_2O and 10 % D_2O (v/v). Conversion of 2OG to succinate was monitored by the triplet at 2.34 ppm for 2OG and the singlet at 2.24 ppm for succinate. Note that the presence of hyCODD led to inhibition of tPHD2-catalysed 2OG conversion to succinate. (Studies with hyNODD were hindered by its limited solubility).*

Supplementary Tables

Table S1. Summary of system setup and calculations. The backbone root mean-square deviation (RMSD) and active site RMSD are reported in Å. The truncated active site is defined as residues Phe366, His374, Asp315, His313, Hyp564, Tyr303, and succinate or 2OG.

Method	System	Co-factor	Time (ns)	System size (atoms)	RMSD ± σ (Å)	Active site RMSD ± σ (Å)
**MD**	tPHD2 *cis*	Succinate	40	51914	1.7 ± 0.3	0.9 ± 0.2
	tPHD2 *trans*	Succinate	40	51914	1.6 ± 0.4	1.0 ± 0.2
	tPHD2 *trans*	2OG	40	51919	1.6 ± 0.3	1.0 ± 0.3
**Refined**	tPHD2 *cis*	2OG	40	51910	1.7 ± 0.5	0.7 ± 0.1
	tPHD2 *trans*	2OG	40	51913	2.0 ± 0.7	0.8 ± 0.2
**Total**			200			

Table S2. QM/MM energy minimisation results. The QM/MM energy function used:[25]

$$E_{TOT}\left(r_{QM}, r_{MM}\right) = E^{QM}\left(r_{QM}\right) + E^{MM}\left(r_{MM}\right) + E^{QMMM}\left(r_{QM}, r_{MM}\right)$$

The results of this minimisation imply that 2OG binding is preferred to that of succinate, consistent with the experimental results.

System	Co-factor	Total system size (atoms)	QM size (atoms)	QM charge	Energy (au)
**tPHD2 *cis***	succinate	51914	32	-1	-658.5
**tPHD2 *trans***	succinate	51914	32	-1	-658.4
**tPHD2 *cis* flip**	2OG	51916	34	-1	-680.1
**tPHD2 *trans* flip**	2OG	51919	34	-1	-680.2

References

1. E. Flashman, L. M. Hoffart, R. B. Hamed, J. M. Bollinger, Jr., C. Krebs and C. J. Schofield, *FEBS J*, 2010, **277**, 4089-4099.
2. L. A. McNeill, E. Flashman, M. R. Buck, K. S. Hewitson, I. J. Clifton, G. Jeschke, T. D. Claridge, D. Ehrismann, N. J. Oldham and C. J. Schofield, *Mol BioSys*, 2005, **1**, 321-324.
3. H. Tarhonskaya, R. Chowdhury, I. K. Leung, N. D. Loik, J. S. McCullagh, T. D. Claridge, C. J. Schofield and E. Flashman, *Biochem J*, 2014, **463**, 363-372.
4. M. A. McDonough, V. Li, E. Flashman, R. Chowdhury, C. Mohr, B. M. Lienard, J. Zondlo, N. J. Oldham, I. J. Clifton, J. Lewis, L. A. McNeill, R. J. Kurzeja, K. S. Hewitson, E. Yang, S. Jordan, R. S. Syed and C. J. Schofield, *Proc Natl Acad Sci USA*, 2006, **103**, 9814-9819.
5. R. Chowdhury, E. Flashman, J. Mecinovic, H. B. Kramer, B. M. Kessler, Y. M. Frapart, J. L. Boucher, I. J. Clifton, M. A. McDonough and C. J. Schofield, *J Mol Biol*, 2011, **410**, 268-279.
6. R. Chowdhury, M. A. McDonough, J. Mecinovic, C. Loenarz, E. Flashman, K. S. Hewitson, C. Domene and C. J. Schofield, *Structure*, 2009, **17**, 981-989.
7. A. Bernheim-Groswasser, R. Shusterman and O. Krichevsky, *J Chem Phys*, 2006, **125**, 084903.
8. K. Bacia, E. Haustein and P. Schwille, *Cold Spring Harbor Protoc*, 2014, **2014**, 709-725.
9. J. A. Aguilar, M. Nilsson, G. Bodenhausen and G. A. Morris, *Chem Commun*, 2012, **48**, 811-813.
10. I. K. Leung, M. Demetriades, A. P. Hardy, C. Lejeune, T. J. Smart, A. Szollossi, A. Kawamura, C. J. Schofield and T. D. Claridge, *J Med Chem*, 2013, **56**, 547-555.
11. R. Chowdhury, I. K. Leung, Y. M. Tian, M. I. Abboud, W. Ge, C. Domene, F. X. Cantrelle, I. Landrieu, A. P. Hardy, C. W. Pugh, P. J. Ratcliffe, T. D. Claridge and C. J. Schofield, *Nat Commun*, 2016, **7**, 12673.
12. M. C. Chan, O. Atasoylu, E. Hodson, A. Tumber, I. K. Leung, R. Chowdhury, V. Gomez-Perez, M. Demetriades, A. M. Rydzik, J. Holt-Martyn, Y. M. Tian, T. Bishop, T. D. Claridge, A. Kawamura, C. W. Pugh, P. J. Ratcliffe and C. J. Schofield, *PloS One*, 2015, **10**, e0132004.

13. E. Flashman, E. A. L. Bagg, R. Chowdhury, J. Mecinovic, C. Loenarz, M. A. McDonough, K. S. Hewitson and C. J. Schofield, *J Biol Chem*, 2008, **283**, 3808-3815.
14. C. E. Stebbins, W. G. Kaelin and N. P. Pavletich, *Science*, 1999, **284**, 455-461.
15. D. Motooka, K. Kawahara, S. Nakamura, M. Doi, Y. Nishi, Y. Nishiuchi, Y. K. Kang, T. Nakazawa, S. Uchiyama, T. Yoshida, T. Ohkubo and Y. Kobayashi, *Pept Sci*, 2012, **98**, 111-121.
16. P. Emsley and K. Cowtan, *Acta Crystallogr D*, 2004, **60**, 2126-2132.
17. W. Humphrey, A. Dalke and K. Schulten, *J Mol Graph*, 1996, **14**, 33-38.
18. M. Buck, S. Bouguet-Bonnet, R. Pastor and A. MacKerell Jr, *Biophys J*, 2006, **90**, L36-L38.
19. W. L. Jorgensen, J. Chandrasekhar, J. D. Madura, R. W. Impey and M. L. Klein, *J Chem Phys*, 1983, **79**, 926-935.
20. B. Brooks, C. Brooks, A. MacKerell, L. Nilsson, R. Petrella, B. Roux, Y. Won, G. Archontis, C. Bartels and S. Boresch, *J Comput Chem*, 2009, **30**, 1545-1614.
21. K. Vanommeslaeghe, E. P. Raman and A. D. MacKerell, *J Chem Inf Model*, 2012, **52**, 3155-3168.
22. K. Vanommeslaeghe, E. Hatcher, C. Acharya, S. Kundu, S. Zhong, J. Shim, E. Darian, O. Guvench, P. Lopes, I. Vorobyov and A. D. Mackerell, *J Comput Chem*, 2010, **31**, 671-690.
23. K. Vanommeslaeghe and A. D. MacKerell, *J Chem Inf Model*, 2012, **52**, 3144-3154.
24. C. G. Mayne, J. Saam, K. Schulten, E. Tajkhorshid and J. C. Gumbart, *J Comput Chem*, 2013, **34**, 2757-2770.
25. J. Hutter, M. Iannuzzi, F. Schiffmann and J. VandeVondele, *Wiley Interdiscip Rev Comput Mol Sci*, 2014, **4**, 15-25.
26. M. Krack and M. Parrinello, *HPC Chem*, 2004, **25**, 29.
27. J. VandeVondele, M. Krack, F. Mohamed, M. Parrinello, T. Chassaing and J. Hutter, *Comput Phys Commun*, 2005, **167**, 103-128.
28. T. Laino, F. Mohamed, A. Laio and M. Parrinello, *J Chem Theory Comput*, 2005, **1**, 1176-1184.
29. A. Laio, J. VandeVondele and U. Rothlisberger, *J Chem Phys*, 2002, **116**, 6941-6947.
30. A. D. Becke, *Phys Rev A*, 1988, **38**, 3098.
31. C. Lee, W. Yang and R. G. Parr, *Phys RevB*, 1988, **37**, 785.
32. S. Goedecker, M. Teter and J. Hutter, *Phys Rev B*, 1996, **54**, 1703.
33. C. Hartwigsen, S. Goedecker and J. Hutter, *Phys Rev B*, 1998, **58**, 3641.

34. F. Maseras and K. Morokuma, *J Comput Chem*, 1995, **16**, 1170-1179.
35. S. E. Wilkins, M. I. Abboud, R. L. Hancock and C. J. Schofield, *ChemMedChem*, 2016, **11**, 773-786.
36. P. Jaakkola, D. R. Mole, Y. M. Tian, M. I. Wilson, J. Gielbert, S. J. Gaskell, A. von Kriegsheim, H. F. Hebestreit, M. Mukherji, C. J. Schofield, P. H. Maxwell, C. W. Pugh and P. J. Ratcliffe, *Science (New York, N.Y.)*, 2001, **292**, 468-472.
37. C. Loenarz, J. Mecinovic, R. Chowdhury, L. A. McNeill, E. Flashman and C. J. Schofield, *Angew Chem Intl Ed*, 2009, **48**, 1784-1787.
